# Disease‐structured *N*‐mixture models: A practical guide to model disease dynamics using count data

**DOI:** 10.1002/ece3.4849

**Published:** 2019-02-05

**Authors:** Graziella V. DiRenzo, Christian Che‐Castaldo, Sarah P. Saunders, Evan H. Campbell Grant, Elise F. Zipkin

**Affiliations:** ^1^ Department of Integrative Biology, College of Natural Science Michigan State University East Lansing Michigan; ^2^ Department of Ecology and Evolution Stony Brook University Stony Brook New York; ^3^ National Audubon Society East Lansing Michigan; ^4^ SO Conte Anadromous Fish Research Lab, Patuxent Wildlife Research Center U.S. Geological Survey Turners Falls Massachusetts; ^5^ Ecology, Evolutionary Biology, and Behavior Program Michigan State University East Lansing Michigan

**Keywords:** Bayesian, Dail–Madsen model, disease ecology, emerging infectious diseases, generalized *N*‐mixture model, hierarchical models, host–pathogen interaction, mark–recapture models, multistate models, occupancy model

## Abstract

Obtaining inferences on disease dynamics (e.g., host population size, pathogen prevalence, transmission rate, host survival probability) typically requires marking and tracking individuals over time. While multistate mark–recapture models can produce high‐quality inference, these techniques are difficult to employ at large spatial and long temporal scales or in small remnant host populations decimated by virulent pathogens, where low recapture rates may preclude the use of mark–recapture techniques. Recently developed *N*‐mixture models offer a statistical framework for estimating wildlife disease dynamics from count data. *N*‐mixture models are a type of state‐space model in which observation error is attributed to failing to detect some individuals when they are present (i.e., false negatives). The analysis approach uses repeated surveys of sites over a period of population closure to estimate detection probability. We review the challenges of modeling disease dynamics and describe how *N*‐mixture models can be used to estimate common metrics, including pathogen prevalence, transmission, and recovery rates while accounting for imperfect host and pathogen detection. We also offer a perspective on future research directions at the intersection of quantitative and disease ecology, including the estimation of false positives in pathogen presence, spatially explicit disease‐structured *N*‐mixture models, and the integration of other data types with count data to inform disease dynamics. Managers rely on accurate and precise estimates of disease dynamics to develop strategies to mitigate pathogen impacts on host populations. At a time when pathogens pose one of the greatest threats to biodiversity, statistical methods that lead to robust inferences on host populations are critically needed for rapid, rather than incremental, assessments of the impacts of emerging infectious diseases.

## 1 INTRODUCTION

Emerging infectious diseases threaten human health, food security, and global biodiversity (Daszak, Cunningham, & Hyatt, 2000; Fisher et al., 2012; Jones et al., 2008). Disease ecologists have used theoretical models (e.g., susceptible, infected, recovered [SIR] models, Kermack & McKendrick, 1927, Anderson & May, 1979; individual‐based models, Briggs, Knapp, & Vredenburg, 2010) to understand and predict pathogen spread and disease dynamics (reviewed in Cooch, Conn, Ellner, Dobson, & Pollock, 2012; Joseph et al., 2013; Langwig et al., 2015). Despite the important theory generated by such models, they are often not practical for guiding disease management because they require large amounts of data to parameterize (Barlow, 1995; Boersch‐Supan, Ryan, & Johnson, 2016; Lloyd‐Smith et al., 2005; Smith et al., 2009). To obtain parameter estimates for disease models, ecologists have relied on mark–recapture which require marking and tracking individuals over time (Table 1; Cooch et al., 2012). However, tracking animals can be difficult and logistically infeasible, especially for cryptic/secretive organisms or in small populations (Conn & Cooch, 2009; Faustino et al., 2004; Harmsen, Foster, & Doncaster, 2011; Lachish, Knowles, Alves, Wood, & Sheldon, 2011; Pryde, O'Donnell, & Barker, 2005). Advanced statistical methods that provide similar inferences as mark–recapture models for unmarked host populations (e.g., populations where individuals are not individually tracked over time) are critically needed to understand disease dynamics, assess pathogen impacts on populations, and develop pathogen mitigation strategies.

**Table 1:** Set of individual, population, and site‐level parameters of interest to disease ecologists

Process and scale	Count vs. detection/non‐detection data		Number of seasons		Parameter
Ecological process	Count	Detection/non‐detection	Dynamic (≥2 seasons)	Single season	
Individual‐level		X	X		Host survival probability
		X	X		Host life expectancy
	X		X		Host reproduction
	X		X		Host immigration
	X		X		Host emigration
	X		X		Transmission probability
		X	X		Expected time to first infection
		X	X		Duration of illness
		X	X		Recovery probability
	X		X	X	Host–pathogen load
		X	X	X	Host infection status
Population‐level	X		X	X	Host population size
	X		X		Host population growth rate
	X	X	X	X	Pathogen prevalence
	X		X	X	Average host infection intensity
Site‐level		X	X	X	Site‐occupancy probability
		X	X		Host extinction probability
		X	X		Pathogen extinction probability
Sampling process		X	X	X	Host detection probability
		X	X	X	Pathogen detection probability
		X	X	X	Observation probability (i.e., seen alive; but unknown disease state)

Note

We summarize the minimal type of observation data needed for inference (i.e., counts/abundance or detection–non‐detection/presence–absence). These quantities may be estimable using multistate mark–recapture, multistate dynamic site‐occupancy models, or disease‐structured *N*‐mixture models. Note that to estimate some parameters, multiple seasons of data are required, while others only require a single season of data.

We highlight the utility of *N*‐mixture models (Dail & Madsen, 2011; Hostetler & Chandler, 2015; Royle, 2004; Zipkin, Sillett, et al., 2014; Zipkin, Thorson, et al., 2014) to study disease dynamics using count data. *N*‐mixture models use data from repeated count surveys of multiple sites within a period of population closure to estimate detection probability of individuals and thus population abundance (Royle, 2004). Our guide is aimed toward disease ecologists with some modeling experience. However, the Supporting Information provides all the tutorials and code needed to implement the models outlined in the main text, including additional variations. Our motivation for this paper is threefold. First, count data are currently underutilized in disease modeling but offer an opportunity to estimate critical parameters (Table 1) from commonly collected data. Second, wider adoption of count data models will enhance field‐based testing of epidemiological theory and enable quantitative evaluation of pathogen mitigation strategies at a time when emerging infectious diseases pose one of the greatest threats to biodiversity. Lastly, recent advances in *N*‐mixture models provide new opportunities to study disease dynamics at large spatial and long temporal scales or in remnant host populations decimated by pathogens, when tracking individuals is not feasible. In cases where count data are unavailable, detection/non‐detection data can be used under a site‐occupancy framework as a cost‐effective alternative for assessing disease dynamics (e.g., extirpation, colonization) and spatial patterns, which can be useful for rapid pathogen assessment and development of mitigation strategies. We focus on *N*‐mixture models and their variants because they provide demographic estimates and detailed disease dynamics (Table 1), whereas detection/non‐detection approaches only estimate a fraction of that information (i.e., infected vs. uninfected sites, colonization/extirpation probability). The Supporting Information is integral to the main text and provides additional details on the development and application of our modeling framework.

## 2 CHALLENGES TO PARAMETER ESTIMATION IN DISEASE MODELING

**Figure 1:**
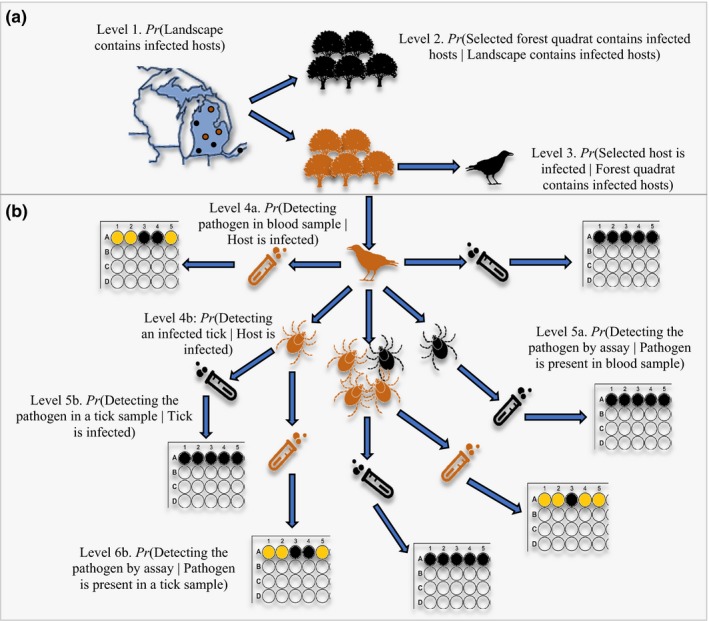
Hierarchical formulation illustrating how imperfect (a) host and (b) pathogen detection manifest in wildlife disease ecology. In this example, we depict *Borrelia burgdorferi*, the causative agent of Lyme disease, infections that are transferred from ticks to birds that inhabit forests. The outer images represent wells of a qPCR plate, and the test tubes represent blood samples. The numbers 1 to 7 illustrate the nested hierarchy of the pathogen within the landscape. Orange shapes indicate infection, including (1) forest sites inhabited by infected birds, (2) infected birds with infected ticks, (3) ticks infected by *B. burgdorferi*, (4) blood samples with *B. burgdorferi *drawn from birds and ticks, and (5) qPCR wells with *B. burgdorferi* DNA. Black shapes indicate no infection. Multiple arrows from a single figure represent repeated samples. To illustrate the concept of nested probabilities, we embed probability statements into the figure. Note that at each level of the hierarchy measurement error may be accommodated using conditional probability statements if count data are subject to sampling bias

Obtaining inference for disease dynamics is challenging because (a) demographic rates (e.g., survival, transmission; Table 1) are difficult to quantify if either population size or recapture rates are low and (b) there are multiple ways in which sampling error affects the observed data (Figure 1). For example, sampling error relevant to disease studies can manifest in two forms: (a) uncertainty of host occurrence or abundance (i.e., imperfect host detection), and (b) uncertainty of pathogen occurrence or abundance (i.e., imperfect pathogen detection; DiRenzo, Campbell Grant, et al., 2018; Lachish, Gopalaswamy, Knowles, & Sheldon, 2012; Miller, Talley, Lips, & Grant, 2012). Historically, sampling error has been either ignored or acknowledged but not modeled (reviewed by McClintock et al., 2010). Yet, failure to account for sampling error can result in biased parameter estimates and thus erroneous management decisions and potentially ineffective disease mitigation strategies (Grant et al., 2017; Langwig et al., 2015; Russell, Katz, Richgels, Walsh, & Grant, 2017).

### 2.1 Estimating demographic rates

Critical life cycle processes, such as birth and death, are not readily observable, which makes estimating demographic rates difficult for most populations. In the context of disease ecology, transmission and recovery events can be cryptic and go unnoticed. For example, pathogen transmission is the product of two events: (a) a contact between an infected and uninfected host and (b) the probability that the infected host transfers the pathogen to the uninfected host. Multistate mark–recapture models have long been used in disease ecology to estimate disease dynamics, but such approaches can be time and labor intensive (Conn & Cooch, 2009; Faustino et al., 2004; Harmsen et al., 2011; Lachish et al., 2011; Pryde et al., 2005; Williams, Nichols, & Conroy, 2002). Mark–recapture studies are therefore often limited to a small number of locations, providing information for a single species or region (e.g., Parmenter et al., 1998, Briggs et al., 2010). The spread and rise of emerging infectious diseases (Jones et al. 2008, Fisher et al., 2012) render mark–recapture methods impractical for rapid pathogen assessment and development of broad‐scale mitigation strategies (Grant et al., 2017; Langwig et al., 2015).

### 2.2 Host detection

Infected and uninfected hosts may have different detection rates based on data collection procedures, and there is no a priori predictable pattern as to which may be easier to observe during sampling. If the detection probability of infected and uninfected hosts differs, then estimates of pathogen prevalence can be biased toward the more detectable host (e.g., Senar & Conroy, 2004; Jennelle, Cooch, Conroy, & Senar, 2007; Conn & Cooch, 2009; Schmidt, 2010; Poulin, 2010). For example, house finches (*Carpodacus mexicanus*) infected by *Mycoplasma gallisepticum* are less detectable than uninfected hosts; not accounting for this bias results in the underestimation of infected individuals in the population (Faustino et al., 2004). In other cases, infected hosts are easier to detect than uninfected hosts, such as European serins (*Serinus serinus*) infected by avian pox (Senar & Conroy, 2004).

### 2.3 Pathogen detection

Pathogen detection probability, which is the probability that a pathogen is detected on a host when it is present, is a significant concern in both veterinary and medical fields and is likely an issue in most sampling and diagnostic methods (reviewed in Enoe, Georgiadis, & Johnson, 2000; Greiner & Gardner, 2000; Mosher et al., 2018; Toft, Jørgensen, & Højsgaard, 2005). The probability of detecting a pathogen on (or within) an infected host is the product of two sequential processes: (a) the sampling of an individual (e.g., blood sample, swab) and (b) laboratory analyses of the sample (e.g., DNA extraction method, qPCR efficiency; Figure 1)*.* Ignoring imperfect detection can result in disease state misclassification, where infected hosts are misclassified as uninfected (i.e., false negatives). This misclassification can lead to biases in estimates of state‐specific survival and transition probabilities and lowers the power to detect differences between survival probabilities of disease states. Across multiple host–pathogen systems, pathogen detection probability using routine laboratory assays shows positive correlations with host infection intensity, such as *Batrachochytrium dendrobatidis* (*Bd*) zoospores on amphibian skin (DiRenzo, Campbell Grant, et al., 2018; Miller et al., 2012); the causative agent of malaria, *Plasmodium* sp., in birds (Knowles et al., 2011; Lachish et al., 2012); the causative agent of tuberculosis, *Mycobacterium bovis*, in cattle (Drewe, Dean, Michel, & Pearce, 2009); and the causative agent of Lyme disease, the bacterium *Borrelia*, in *Ixodes uriae* ticks (Gómez‐Díaz, Doherty, Duneau, & McCoy, 2010). This suggests that infected individuals with low infection intensities are likely to be misclassified as uninfected in many systems using a variety of methods.

## 3 DISEASE‐STRUCTURED *N*‐MIXTURE MODELS

The *N*‐mixture model (Royle, 2004) is a type of state‐space model where the true state of the system (i.e., animal abundance) is assumed to be imperfectly observed during the sampling process. *N*‐mixture models attribute observation error to imperfect detection of individuals (i.e., false negatives) which can be estimated from repeated surveys of a site over a period in which the population is closed to demographic changes. A site can be defined as a spatial location, an individual, or a sample; a repeated survey can be defined as temporal or spatial replicates, or resampling of an individual.

In this section, we introduce the disease‐structured *N*‐mixture model framework for closed (single season) and open (multiseason) populations (see Supporting Information for additional details). Disease‐structured *N*‐mixture models provide a rapid, inexpensive, and efficient approach for estimating demographic rates, while accounting for imperfect host and/or pathogen detection (e.g., Dail & Madsen, 2011; Zipkin, Sillett, et al., 2014; Zipkin, Thorson, et al., 2014; Hostetler & Chandler, 2015; Zhao, Royle, & Boomer, 2017; Brintz, Fuentes, & Madsen, 2018). Using data collected across time and space, disease‐structured *N*‐mixture models can estimate state‐dependent abundance and related demographic rates (e.g., survival, recruitment, immigration), as well as transition probabilities between disease states (Table 1).

Generalized *N*‐mixture models have recently been applied to two disease‐specific case studies (Brintz et al., 2018; DiRenzo, Zipkin, et al., 2018). Brintz et al. (2018) estimate an increase in the number of chlamydia cases in Oregon over time and highlight how imperfect pathogen detection may be due to latency of the infection or possibly hard‐to‐reach populations, including drug users, sex workers, and others having the infection. In a different model formulation, DiRenzo, Zipkin, et al. (2018) evaluated support for three host–pathogen coexistence hypotheses (i.e., source‐sink, eco‐evolutionary rescue, and spatial variation in pathogen transmission) in a Neotropical amphibian community decimated by *Bd* in 2004. They found that the primary driver of host–pathogen coexistence was eco‐evolutionary rescue, as evidenced by similar amphibian survival and recruitment rates between infected and uninfected hosts.

### 3.1 Single‐season closed population model

We outline the general modeling framework for a closed population disease‐structured *N*‐mixture model to estimate the abundance of infected and uninfected hosts using one of the most frequently collected data types: host and pathogen counts. The goal is to estimate pathogen prevalence and site‐level abundance of infected and uninfected hosts, while accounting for both imperfect host and pathogen detection. In the single‐season study design, we assume that individual sites (e.g., survey plots or transects) are sampled on multiple occasions (survey replicates) during a time frame when the host population is closed to changes (i.e., birth, death, immigration, emigration, disease transmission). The observed number of infected and uninfected hosts is counted at each site during each survey event. Each observed host is assigned a disease state (infected or not infected), based on diagnostic symptoms of disease or laboratory analysis (e.g., swabs, blood, or tissue samples), and used to calculate the average infection intensity at site *i* during survey *j*. We assume that a fraction of infected hosts are misidentified as uninfected because of low pathogen infection intensities (e.g., DiRenzo, Campbell Grant, et al., 2018; Lachish et al., 2012; Miller et al., 2012), but misidentification may also be unrelated to infection intensity and attributable to other factors (e.g., field sampling and laboratory diagnostic testing).

We specify the observation model to account for imperfect host and pathogen detection during the sampling process by assuming that pathogen detection is related to infection intensities (e.g.,DiRenzo, Campbell Grant, et al., 2018; Lachish et al., 2012; Miller et al., 2012). We denote *g_s,i,j_* as the number of hosts detected in each disease state *s* (where *s* = 1 for uninfected and *s* = 2 for infected) at site *i* during survey replicate *j. *We assume that there are a number of misidentified infected hosts, *m_i,j_* (i.e., the number of infected hosts classified as uninfected when they are actually infected). The corrected number of detected uninfected and infected hosts, *y_s,i,j_*, in disease state *s *at site *i* during survey *j* is part of a deterministic relationship between the number of observed hosts, *g_s,i,j_*, and the number of misspecified hosts, *m_i,j_*:

(1) $$g_{1,i,j}=y_{1,i,j}+m_{i,j},$$

(2) $$g_{2,i,j}=y_{2,i,j}-m_{i,j}.$$

We assume that the number of misspecified individuals, *m_i,j_*, is a binomial random variable dependent on the average pathogen detection probability for hosts at site *i *during survey replicate *j*, *θ_i,j_*:

(3) $$m_{i,j}∼\text{bin}\left(y_{2,i,j},1-\theta_{i,j}\right).$$

To estimate *θ_i,j_, *we model the relationship between pathogen detection probability and average site‐specific pathogen infection intensity, Z*_i,j_*: logit(*θ_i,j_*)* = δ*0* *+* δ*1 Z*_i,j_*, where *δ*0 and *δ*1 are the intercept and slope parameters on the effect (see Supporting Information for details). *Z_i,j_* is estimated using a lognormal distribution:

(4) $$Z_{i,j}∼\log\text{normal}\left(\log\left(\mu_{i}+0.001\right),\sigma^{2}\right)$$

where, *µ_i_*is the average infection intensity at site *i *and *σ* ^2^ is the variation in infection intensity among individuals at site *i*. The observed data, *x_i,j_*, is then modeled as a lognormal distribution with mean Z*_i,j_* and sampling error *σ_e_* ^2^:

(5) $$x_{i,j}∼\log\text{normal}\left(\log\left(Z_{i,j}+0.001\right),\sigma_{e}^{2}\right).$$

The parameters *δ*0, *δ*1, *µ_i_*, and *σ* ^2^ are estimable by either (a) collecting multiple (>1) samples for diagnostic analysis from each observed host or (b) repeated diagnostic runs for individual samples, to explicitly model the relationship between pathogen infection intensity and pathogen detection probability. In cases where such data are unavailable, information obtained from outside studies can be used. The relationship between pathogen intensity and detection probability has been established for many disease systems (Drewe et al., 2009; Gómez‐Díaz et al., 2010; Knowles et al., 2011; Lachish et al., 2012; Miller et al., 2012, DiRenzo, Campbell Grant, et al., 2018), which can be readily translated into informative priors (e.g., DiRenzo, Zipkin, et al., 2018).

We use the corrected number of uninfected and infected hosts *y_s,i,j_* in our model to estimate the true number of uninfected and infected hosts at each site *i*, *N_s,i_, *with the binomial distribution*:*

(6) $$y_{s,i,j}∼\text{bin}\left(N_{s,i},p_{s,i,j}\right),$$

where *p_s,i,j_* is the host detection probability in disease state *s* at site *i* during survey *j*.

We model true host abundance, *N_s,i_,* in disease class *s* and site *i* using a count distribution, such as the Poisson:

(7) $$N_{s,i}∼\text{Poisson}\left(\lambda_{s}\right)$$

where *λ_s_* is the expected host abundance for disease state *s* across all sites, which can also be modeled with site‐level habitat covariates using a log‐link function. We use the Poisson distribution but others such as the zero‐inflated Poisson or negative binomial, can be used in the case of excess zeros or when data are skewed (Kéry & Schaub, 2012).

Total host abundance at a site *i* is the sum of the latent number of infected and uninfected hosts: ${\tilde{N}}_{i}=N_{1,i}+N_{2,i}$. Site‐level pathogen prevalence, *P_i_* is then calculated as:

(8) $$P_{i}=\frac{N_{2,i}}{{\tilde{N}}_{i}}$$

### 3.2 Multiseason open population model

**Figure 2:**
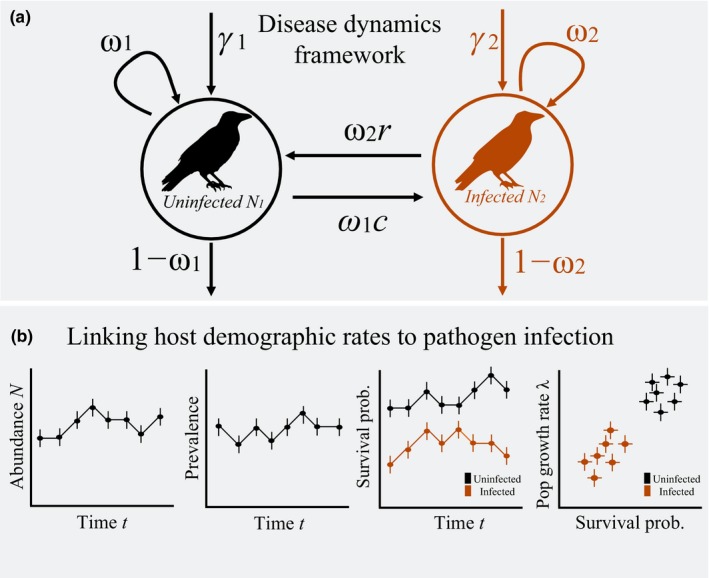
Disease dynamics of a host population governed by (a) the uninfected and infected sub‐populations (*N*_1_ and *N*_2_, respectively) that experience state transitions (i.e., *c* = infection, *r* = recovery), recruitment (*γ*), and survival (*ω*). This framework has traditionally been used in mark–recapture models, but recent advancements in unmarked data models allow for a similar parameter estimation. Parameters are defined in Table 1. To study the link between host demographic rates and pathogen infection, we can (b) correlate the estimates of annual population growth rates with those of estimated demographic rates in both infected and uninfected states

In the multiseason formulation of the disease‐structured *N*‐mixture model, our goal is to estimate the number of individuals (e.g., infected, uninfected), demographic parameters (e.g., survival, recruitment rates), and disease dynamics (i.e., transmission, recovery probabilities; Figure 2). Using a robust multiseason study design, sites are repeatedly surveyed during multiple primary sampling periods (e.g., seasons or years; Dail & Madsen, 2011; Zipkin, Sittell, et al., 2014; Zipkin, Thorson, et al., 2014). We assume that the population is open to changes in abundance via birth, survival, immigration, and changes in disease states between primary sampling periods. Within each primary sampling period, sites are repeatedly sampled during a period of population closure, and all hosts are counted and assigned to a disease state (i.e., infected or not infected) as described in the closed population model. The observation model linking the observed survey data (i.e., repeated counts of infected and uninfected hosts at sites) for the open population model is the same as in the closed population model with the only difference being that the data and true latent abundance of hosts in disease state *s *at each site *j* during primary season, *N_s,i,t_*, are all also indexed by season/year *t*.

We model abundance for each disease state *s* in the first year, *N_s,i,1_*, of sampling using a count distribution, such as a Poisson:

(9) $$N_{s,i,1}∼\text{Poisson}\left(\lambda_{s}\right),$$

where expected host abundance, *λ_s_*, differs by disease state *s*. Expected abundance could also change by location if site‐level covariates (indexed by *i*) are included. We model subsequent seasons (*t* ≥ 2) by considering the number of hosts at each site *i* that survive in disease state (*S*), transition between disease states (*T*) and are recruited via immigration and reproduction (*G*).

To model the number of hosts that survive from season *t* − 1 to *t* at site *i*, we define parameter *ω_s_* as the disease state‐specific apparent survival probability for an uninfected (*s = *1) and infected (*s* = 2) hosts, such that:

(10) $$S_{s,i,t}∼\text{bin}\left(N_{s,i,t-1},\omega_{s}\right).$$

We specify the number of hosts that transition from disease state *s* to *ss* at site *i* from season *t* − 1 to *t* (*T_s_*_(ss)_ *_,i,t_*) based on site‐specific transmission risk (*c_i,t_*) and recovery probability (*r_i,t_*):

(11) $$T_{1(2),i,t}∼\text{bin}\left(S_{1,i,t},c_{i,t}\right),$$

(12) $$T_{2(1),i,t}∼\text{bin}\left(S_{2,i,t},r_{i,t}\right).$$

With this specification, hosts must first survive with the probability associated with their disease state *ω_s_* in season *t* − 1 before transitioning to another disease state. Finally, we model the number of hosts gained, *G_s,i,t_,* to each disease state *s* at site *i* from season *t* − 1 to *t*:

(13) $$G_{s,i,t}∼\text{Poisson}\left(\gamma_{s,i,t}\right),$$

where *γ_s,i,t_* is the expected number of uninfected (*s* = 1) and infected (*s = *2) hosts recruited (either by immigration or reproduction) to site *i* between seasons.

The state‐specific host abundance, *N_s,i,t_*, for disease state *s* at site *i* during season *t *is then the sum of the number of hosts that are gained at a site, survive at a site, and remain there, and those that transition into disease state *s* minus those that transition out of disease state *s*:

(14) $$N_{s,i,t}=G_{s,i,t}+S_{s,i,t}+T_{\text{ss}(s),i,t}-\;T_{s(\text{ss}),i,t}$$

### 3.3 Assumptions and limitations

While we highlight the utility and potential for the use of *N*‐mixture model to studying disease dynamics, the benefits of cheaper sampling designs (i.e., count data compared to mark–recapture) comes at inferential costs, such as restrictive assumptions and less power (Table 2; Barker, Schofield, Link, & Sauer, 2018; Link, Schofield, Barker, & Sauer, 2018). Like all statistical models, *N*‐mixture models are an oversimplification of the biological world, but they provide the opportunity to extrapolate demographic information from count data and account for sampling error. Kéry (2018) concluded that parameter identifiability problems associated with *N*‐mixture models arise when the number of sites sampled is small, but that most models are well identifiable.

**Table 2:** Assumption violations, problems, and solutions to *N*‐mixture models

Violation	Why is it a problem?	What can be done?	Citation
Double counting	When less than one animal in twenty is double counted, then the model biases estimates of abundance by 21%	Use *N*‐mixture model for relative abundances or allocate additional resources for mark–recapture data	Link et al. (2018)
Unmodeled variation in population size over time	Estimation of average abundance is biased, bias increases as the proportion of variation in population size that occurs among sites decreases	Use *N*‐mixture model for relative abundances or allocate additional resources for mark–recapture data	Link et al. (2018)
Unmodeled variation in detection probability over time	2% variation in detection results in 19% to 21% additional bias in estimation of average abundance	Use *N*‐mixture model for relative abundances or allocate additional resources for mark–recapture data	Link et al. (2018)
Unmodeled variation in detection probability over time	Alternative models are indistinguishable, and no reliable estimate for abundance can be obtained	Use *N*‐mixture model for relative abundances or allocate additional resources for mark–recapture data	Barker et al. (2018)
When detection probability and number of sampling occasions are small	An infinite estimate of abundance can arise	Use a sample covariance as a diagnostic test to identify this problem	Dennis, Morgan, and Ridout (2015)

Failure to meet modeling assumptions for structured *N*‐mixture models (e.g., disease, age, sex, size, etc.) will likely result in similar problems to single‐season *N*‐mixture models (Table 2), but such models have not yet been explicitly evaluated. Structured *N*‐mixture models may be more susceptible to unidentified parameter biases, depending on circumstances, because of their complicated framework. Assumption violations may also have cascading effects. For example, if the closure assumption were violated such that there was high immigration of infected hosts within a primary sampling period, estimates of disease transmission and infected host survival probability could be overestimated, and recovery and uninfected host survival probability could be underestimated. Likewise, estimates of infected and uninfected host abundance would be biased, overestimating numbers of infected hosts, and therefore, pathogen prevalence and average infection intensity. Structured *N*‐mixture models are also likely to require a greater number of sites, more repeated surveys, and a larger number of primary seasons in order for parameter identifiability. Thus, a wider breadth of count data is required to compensate for the lack of depth of demographic information that is otherwise present in mark–recapture data.

## 4 FUTURE DIRECTIONS

Emerging infectious diseases are challenging to forecast, which impedes development of optimal pathogen control strategies (Daszak et al., 2000; Jones et al., 2008; Fisher et al., 2012; but see Russell et al., 2017). One of the primary goals for management of emerging infectious diseases is to minimize pathogen spread and their impacts on host populations (Bielby, Cooper, Cunningham, Garner, & Purvis, 2008; Kilpatrick, Daszak, Goodman, Rogg, et al., 2006; Kilpatrick, Daszak, Jones, Marra, & Kramer, 2006; Kilpatrick, Kramer, Jones, Marra, & Daszak, 2006; Langwig et al., 2015; Smith, Waller, Russell, Childs, & Real, 2005). Given the typically small population sizes of wildlife disease ecology studies, *N*‐mixture models can be a powerful tool to estimate disease dynamics, especially when combined with other sources of information (e.g., informative priors) or data (e.g., mark–recapture). Below, we suggest potential avenues of research to further exploit unmarked datasets for disease analyses.

### 4.1 False positives

Ignoring false positives also results in disease state misclassification, where uninfected hosts are misclassified as infected (i.e., false positive). False positives (i.e., incorrectly diagnosing uninfected hosts as infected) can occur during pathogen diagnostic tests because of cross‐contamination of samples or lack of diagnostic test specificity. Consideration of false positives in disease ecology is scarce (but see Abad‐Franch, Valença‐Barbosa, Sarquis, & Lima, 2014) as most studies assert that either strict protocols were used to prevent cross‐contamination or that negative controls on PCR plates were used to identify cases where false positives occurred (e.g., Lachish et al., 2012; Miller et al., 2012; Schmidt, Kéry, Ursenbacher, Hyman, & Collins, 2013; Zelé et al., 2014). However, strict protocols alone may not be enough to eliminate false positives. For example, false positives can occur from carryover of amplified DNA sequences, reagent contamination, positive displacement by pipettes, and aerosol effects (Kwok, 1990). Modeling approaches that estimate both false negatives and false positives could accommodate ambiguous state assignment by supplying prior information on the probability of a false positive (e.g., Miller et al., 2011). The prior information can take the form of an inequality constraint (e.g., false negatives < false positives; see Royle & Link, 2006) or a prior distribution informed by a priori knowledge, expert opinion, or data (Chambert, Miller, & Nichols, 2015; Miller et al., 2011). To date, there have only been detection/non‐detection models of false positives (Chambert et al., 2015; Miller et al., 2011). If *N*‐mixture models were combined with false‐positive models, then that would aid in discerning double counts (see Table 2 for consequences of double counts).

### 4.2 Spatially explicit models

An emerging frontier for *N*‐mixture models is spatially explicit dynamic models (Zhao et al., 2017), in which emigration and immigration are separated from survival and reproduction, respectively. Spatially explicit models have the advantage of providing biologically realistic models across heterogeneous space. Spatial heterogeneity can be added into models through covariates, with random spatial effects (e.g., Yackulic et al., 2012), or by making assumptions about the ability of individuals to move between sites (e.g., assuming that movement can only occur among adjacent habitat patches; Zhao et al., 2017). Within a disease context, spatially explicit models could be used to identify spatial metapopulations or habitats that serve as pathogen hotspots (i.e., areas where pathogen survival and transmission are high) or host refugia (i.e., areas where host survival is high and pathogen transmission is low; Paull et al., 2012). In this case, conservation managers can target disease prevention to specific metapopulations or habitat patches rather than an entire region.

### 4.3 Integration of untapped data sources

Count data can be combined with other data types (e.g., mark–recapture data, opportunistic presence‐only data) to model disease dynamics. Recently developed approaches, such as integrated population models and integrated distribution models, combine different types of data including count, detection–non‐detection, mark–recapture data, or opportunistic presence only (e.g., Koshkina et al., 2017; Morris, Reich, Pacifici, & Lei, 2017; Pacifici et al., 2017; Zipkin et al., 2017), improving parameter accuracy and precision, and thus inferences on population‐level processes. New modeling frameworks are regularly developed to take advantage of cheaply collected data to estimate demographic rates. For example, the dynamic *N*‐occupancy model uses only detection–non‐detection data to estimate abundance, population gains (i.e., via immigration and reproduction), and apparent survival probabilities (Rossman et al., 2016). Extending such models to accommodate disease structures might be useful in the integration of multiple data types, provided that certain assumptions and data requirements are met.

Applying *N*‐mixture models to long‐term and/or spatially expansive datasets may allow for assessments of historic patterns of disease dynamics and predictive capacity to forecast impacts of novel pathogen invasions. Citizen science programs, such as the FeederWatch project (http://feederwatch.org/), Saving Salamanders with Citizen Science (http://www.amphibians.org/), ZomBee Watch (https://www.zombeewatch.org/), and Monarch Health (http://monarchparasites.org/), have the capacity to contribute valuable data to such analyses. Combining citizen science data with data obtained from designed, researcher‐collected surveys is likely to yield more precise estimates of demographic and disease parameters than can be obtained solely from the individual sources (e.g., van Strien, Swaay, & Termaat, 2013).

## 5 CONCLUSIONS

The rise of emerging infectious diseases requires rapid creation of disease mitigation programs (e.g., Langwig et al., 2015). Yet, to develop effective preventions and controls, wildlife managers need estimates of disease prevalence, pathogen infection intensity, transmission, and recovery rates—all of which may be biased by sampling error. The *N*‐mixture modeling framework that we outline here can be modified for specific control scenarios and can additionally accommodate outside sources of data—including expert opinion, borrowed information from better‐studied disease systems, and laboratory or pilot studies (Russell et al., 2017). Model extensions can include more complicated disease structures such as multiple host species, multiple pathogen infections, age structure, stage structure, or heterogeneity in rates, although inevitably more data will be needed to estimate additional parameters in such models. The template that we provide (including code within the Supporting Information) can be customized based on specific study objectives. Unmarked data offer excellent opportunities to understand complex host–pathogen interactions, bridging the long‐standing gap between disease ecology and theory‐based wildlife disease management.

## CONFLICTS OF INTEREST

The authors declare no conflicts of interest.

## AUTHOR CONTRIBUTIONS

GVD wrote the first draft of the manuscript. All authors contributed to the ideas, editing, and review of subsequent drafts.

## Data Availability

This manuscript does not use any data, but all code and R scripts to simulate data can be found in the Supporting Information or online on the Github repositories of Grace89 (https://doi.org/10.5281/zenodo.2223304).
