# Supplementary material for: Disease‐structured N‐mixture models: A practical guide to model disease dynamics using count data
Source: Ecol Evol. 2019 Feb 5;9(2):899–909. doi: 10.1002/ece3.4849 (PMC6362444; doi:10.1002/ece3.4849)
Supplement: Supplementary file 1 [file ECE3-9-899-s001.pdf]

Foreword

S1: Glossary

S2: Mark-recapture models in disease ecology

S2.1: History of mark-recapture models in disease ecology

S2.2: Multistate mark-recapture model

S2.3: Parameters of interest

S2.4: Model outline

S3: Model assumptions

S3.1: Unmarked data assumptions

S3.2: Mark-recapture model assumptions

S4: Disentangling sampling and diagnostic errors

S4.1: Parameters of interest

S4.2: Model outline

S4.3: Simulating data

S4.4: JAGS code

S4.5: Analyzing the data

S5: Disease-structured N-mixture model (single season)

S5.1: Table

S5.2: Model outline

S5.3: Simulating data

S5.4: JAGS code

S5.5: Analyzing the data

S6: Disease-structured generalized N-mixture model (multi-season)

S6.1: Table

S6.2: Simulating data

S6.3: JAGS code

S6.4: Analyzing the data

S7: Steps for designing a study and modeling a system

S8: Common interfaces and programs to run models

Table

S9: References

Foreword

Note: the simulations in this appendix generally represent best-case scenarios (i.e., lots of surveys and sites; high abundance and detection probability) to provide the reader with the necessary tools for data analysis. We encourage readers to use this code as a tutorial. Interested readers can use this appendix as a template to create their own power analysis for a specific sampling design, adjusting parameter values and the number of surveys/sites to fittheir system. Other papers that examine tradeoffs in sampling design for particular parameter estimates in unmarked data models include MacKenzie & Royle 2005, Zipkin et al. 2014*a*, and Kéry 2018.

S1: Glossary

Abstract- This section serves to aid the reader in understanding words or phrases used throughout the manuscript. The definitions used here are consistent with the most cited literature on the topics of site-occupancy models, *N*-mixture models, and disease ecology.

Closed population: A population that does not experience births, deaths, immigration, or emigration.

Conditional probability: The probability that some event A given the occurrence of some other event B will occur, written as: \(\textrm{Pr}(A \mid B)\).

Convergence: The parameters have reached a stationary distribution for the posterior. Convergence can never be proven but visual (e.g., well-mixed chains) and numeric (e.g., Rhat) checks are adequate.

Detection probability: Under the occupancy model framework, this refers to the probability that the focal species is detected at a site given that it is present and available for capture/observation. Under the N-mixture model framework, this refers to the probability of detecting an individual at a site given that it is present and available for capture/observation.

Disease ecology: The study of interactions between infectious agents (i.e., micro- or macro-parasites) and their hosts, including transmission dynamics; factors underlying patterns of variation in infection; the effects of an infectious agent on host behavior, population dynamics

and community structure; and coevolutionary relationships between hosts and infectious agents.

Enzootic: A disease that regularly affects animal populations. Non-human equivalent of endemic.

Epizootic: A disease that is temporarily extremely prevalent and widespread in an animal population. Non-human equivalent of epidemic.

False negative: Incorrectly identifying the absence of a species given the species is truly present.

False positive: Incorrectly identifying the presence of a species given the species is truly absent.

Hierarchical model: A sequence of related models ordered by their conditional probability structure, meaning that these models have one or more intermediate models/levels/stages involving a latent variable or random effect.

Host: The organism from which an infectious agent obtains its nutrition or shelter.

Infection intensity: Number of infectious agent cells (e.g., spores, infective stage) present on an infected individual at a particular time.

Latent state: The true unobserved state, generally inferred from mathematical relations.

Mark-recapture models: Also referred to as “marked data models”. These models require encounter histories composed of detection/non-detection data from i marked hosts over t sampling occasions.

Macroparasite: An infectious agent that grows but does not multiply in the host, producing infective stages that are released to infect new hosts. Macroparasites of animals mostly live on the body or in the body cavities (e.g., gut); in plants, they are generally intracellular.

Measurement error: Sampling error when quantifying host abundance or infection intensity of infected individuals.

Microparasite: A small, often intracellular infectious agent that multiples directly within the host.

Nested probability statement: A probability statements that depends on the output of a probability statement. See Fig. 1 in the main text for an example.

Parasite: An organism that obtains its nutrients from one or a few hosts, normally causing harm but not causing death immediately.

Pathogen: The term that may be applied to any parasite that gives rise to a disease.

Prevalence: Proportion of positive cases in a host population at a particular time.

Process variance: The variation in a biological processes of interest.

Observation variance: The uncertainty that results from imperfect observations, including sampling error in estimates.

Open population: A population that experiences births, deaths, immigration, or emigration

Rhat: A formal convergence test criterion comparing the among- and the within-chain variance in an ANOVA fashion; values around 1 suggest the absence of a "chain effect" and therefore convergence. Often, values of 1.1 or 1.2 are taken as the upper limits of Rhat-values that indicate convergence.

Transmission: The process by which susceptible hosts acquire infectious agents. The rate of transmission depends on the contact rate between hosts, the probability that a contact is with an infectious host, and the transmission probability given contact.

Unmarked data model: A type of state-space model in which the true state of the system (i.e., occupancy or abundance) is imperfectly observed. We are limiting the definition of unmarked models to include: site-occupancy model, dynamic occupancy model, N-mixture model, and generalized N-mixture model. These unmarked data models specifically attribute observation error to imperfect detection, as calculated from replicate site surveys over a period of population closure, which leads to false negatives.

S2: Mark-recapture models in disease ecology

Abstract- In this section, we aim to provide a quick overview of the history of mark-recapture models in disease ecology, an outline for the multi-state mark-recapture model, a table with a list of state variables, observed data, and parameters of interest, and a model outline. More information and code on multistate mark-recapture models can be found in Kéry & Schaub (2012).

S2.1: History of mark-recapture models in disease ecology

Traditionally, disease ecologists have used multistate mark-recapture models to study disease dynamics in wild host populations (Table 1; reviewed by Cooch et al. 2012). These models require encounter histories composed of detection/non-detection data from *i* marked hosts over *t* sampling occasions in one of *s* discrete disease states. Individual model parameters can be modeled as linear functions of one or more covariates of interest using the appropriate link functions and design matrices. Construction of a multistate mark-recapture model analogous to a classic compartment disease model (e.g., SIR model) proceeds naturally, where transitions between disease states are determined by the joint probability of surviving and moving between disease states (see Fig. 2 main text).

The classic multistate mark-recapture model accounts for state-specific differences in host detection probability and assumes that states are assigned without error, ignoring imperfect pathogen detection probability and partial observations (i.e., animal seen alive but state unknown). To address the latter issue, Conn & Cooch 2009 developed a multistate mark-recapture model using a hidden Markov modeling framework to incorporate data from encounters of unknown disease state. This approach allows ecologists to include records where state information is missing, instead of censoring data.

One of the biggest differences between marked and unmarked data models is the scale of data collection and inference. For example, marked models take data on individuals, and inference is made on the individual; whereas the data collection and inference scales depend on the type of unmarked data model. For instance, occupancy models take data and make inference on the 'site' level (e.g., habitat patch); but the generalized N-mixture model takes data on at the site level and make inference for individuals within a site.

S2.2: Multistate mark-recapture model

Multistate mark-recapture models have long been used in disease ecology to describe disease dynamics. Here, we present a multistate Jolly-Seber model to estimate host survival, entry, and disease state transition probabilities for a host population across several seasons while accounting for imperfect host detection. We used the multistate formulation of the Jolly-Seber model, which includes both state and observation processes (Kéry & Schaub 2012). The state process describes the true disease state of an individual where state *s* is described as not entered, uninfected, infected, and dead, and assumes that an individual moves between these 4 disease states over a finite number of sampling occasions *t*. For any given individual, the successive disease state is described by a discrete first-order hidden Markov model, where the probability of an individual transitioning to or remaining in a disease state from time *t-1* to *t* only depends on the current state at time *t-1*. To estimate true abundance, we use a data augmentation method. We augment the observed data set *y* with a large number of all-zero

capture-histories, resulting in a larger data set of fixed dimension *Q*, where *Q* is much greater than *N*, the true population size. By augmenting the observed data set *y*, we account for individuals never observed during surveys but that were likely present.

S2.3: Parameters of interest

List of state variables, observed data, and parameters of interest along with their definitions.

Quantity	Type	Definition
$z_{i,t}$	state variable	The true state of host *i* at sampling occasion *t*
$y_{i,t}$	observed data	The observed state of host *i* at sampling occasion *t*
ϕ_{2}	parameter	The survival probability of uninfected hosts
ϕ_{3}	parameter	The survival probability of infected hosts
γ_{1}	parameter	The probability that a host does not enter the population at time *t*
γ_{2}	parameter	The probability that a host enters the population uninfected at time *t*
γ_{3}	parameter	The probability that a host enters the population infected at time *t*
β_{IU}	parameter	The probability of transitioning from infected to uninfected from *t-1* to *t*
β_{UI}	parameter	The probability of transitioning from uninfected to infected from *t-1* to *t*
p_{2}	parameter	The probability of detecting an uninfected host
p_{3}	parameter	The probability of detecting an infected host

S2.4: Model outline

We denote the true but, unknown, disease state of individual *i* at sampling occasion *t* as $z_{i,t}$, where $z_{i,t} = 1\ldots4$ and represents the states “not entered”, “uninfected”, “infected”, or “dead”, respectively. To estimate monthly survival (ϕ), recruitment (γ), and transition (β) rates, we used the transition matrix ψ, where the cells represent the probabilities of moving from a row state to a column state from time *t-1* to time *t* for host *i*.

$$\psi = \begin{array}{r} \\ \text{not entered} \\ \text{uninfected} \\ \text{infected} \\ \text{dead} \end{array} \begin{array}{c} \begin{array}{cccc} \text{not entered} & \text{uninfected} & \text{infected} & \text{dead} \end{array} \\ \left[\begin{array}{cccc} \gamma_1 & \gamma_2 & \gamma_3 & 0 \\ 0 & \phi_2(1-\beta_{UI}) & \phi_2\beta_{UI} & 1-\phi_2 \\ 0 & \phi_3\beta_{IU} & \phi_3(1-\beta_{IU}) & 1-\phi_3 \\ 0 & 0 & 0 & 1 \end{array} \right] \end{array}$$

The “not entered” category consists of individuals that are not part of the population yet, where the parameters γ_{2} and γ_{3} are the state-specific entry probabilities for uninfected and infected hosts from *t-1* to *t*, i.e., the probability that a host in state *s* enters the population at time *t*.

The γ parameters are the probabilities that an available individual in *M* (the data augmentation) enters the population at occasion *t*. Importantly, γ refers to available individuals, that is, to those in *M* that have not yet entered. The entry process is thus a removal process; over time, fewer and fewer individuals will be in the state “not yet entered” and thus available to enter the population. Thus, γ is the removal entry probability. Depending on the type of Jolly-Seber model parameterization used (i.e., Restricted Dynamic Occupancy Model, Superpopulation parameterization, Multistate parameterization), there are different ways to calculate the “entry probability” and the number of individuals entering each season.

The parameter ϕ_{2} and ϕ_{3} are the state-specific survival probability for uninfected and infected hosts from *t-1* to *t*, while the parameters β_{UI} and β_{IU} are the infection and recovery probabilities, respectively. In other words, if host *i* survives from *t-1* to *t*, it can become infected if they were uninfected at *t-1*, or recover from infection if there were infected at time *t-1*, with probabilities β_{UI} and β_{IU}, respectively. Each parameter must be between 0 and 1, and each row of the matrix must sum to 1. For survival and transition this is easy. However, for the removal entry probability, γ, this is less straightforward. One solution is to choose a Dirichlet prior for $[\gamma_{1}, \gamma_{2}, \gamma_{3}]$ to ensure that these parameters sum to 1.

Because there are more than two possible true and observed states, we use the categorical distribution to model the transition from one state to another for host *i* each sampling occassion, *t*:

$$z_{i,t} \mid z_{i,t-1} \sim \textrm{categorical}(\psi_{z_{i,t-1},1:4}),$$

where ψ is the transition matrix defined above. Note that the argument of the categorical distribution is a vector of length 4; that is, it is the row of the matrix ψ corresponding to the state *s* of host *i* in time step *t-1*.

To estimate host recapture probabilities, we use a second transition matrix π, which determines the probabilities of the possible observation outcomes (columns) for the true state of each captured individual (rows). We do not assume partial observations or state misclassifications and the observed states are “seen uninfected”, “seen infected”, and “not seen”.

$$\pi = \begin{array}{r} \\ \text{not entered} \\ \text{uninfected} \\ \text{infected} \\ \text{dead} \end{array} \begin{array}{ccc} \text{seen unifected} & \text{seen infected} & \text{not seen} \\ \hline 0 & 0 & 1 \\ p_2 & 0 & 1 - p_2 \\ 0 & p_3 & 1 - p_3 \\ 0 & 0 & 1 \end{array}$$

We use the categorical to model the observed state $y_{i,t}$ as a function of the true state:

$$y_{i,t} \mid z_{i,t} \sim \textrm{categorical}(\pi_{z_{i,t},1:3}),$$

where π is the observation matrix we just defined. Similarly as before, the argument of the categorical distribution is a vector of length 3; that is, it is the row of the matrix π corresponding to the true state of individual *i* in time step *t*.

S3: Model assumptions

Abstract- In this section, we aim to list the assumptions that need to be met for a variety of different mark-recapture, site-occupancy, and N-mixture models. Violations of these assumptions can lead to parameter bias, misleading inference and conclusions.

S3.1: Unmarked data assumptions

Site-occupancy model assumptions (MacKenzie et al. 2002) and *Multistate occupancy model assumptions* (MacKenzie et al. 2005; also see Bailey et al. 2014):

1. The occupancy status of a site does not change over the course of sampling (i.e., closed population)

2. The probability of occupancy is the same across all sites or any heterogeneity is modeled using covariates

3. The probability of detection is the same across all sites or any heterogeneity is modeled using covariates

4. Detection histories at each site are independent

5. Sites are a random sample of the population

Dynamics site-occupancy model assumptions (Royle & Kéry 2007)

1. The occupancy status of a site does not change over the course of sampling (i.e., closed population)

2. The occupancy status of a site may change between seasons, i.e., populations are “open” to extinction and colonization

3. The probability of occupancy, extinction, and colonization is the same across all sites or any heterogeneity is modeled using covariates

4. The probability of detection is the same across all sites or any heterogeneity is modeled using covariates

5. Detection histories at each site are independent

6. Sites are a random sample of the population

N-mixture model assumptions (Royle, 2004):

1. The ecological state (i.e, abundance) is constant during the period over which replicate surveys are conducted (i.e., closure assumption)

2. Detection probability is constant across individuals present during a survey

3. The distributions of abundance and detection are adequately described by the chosen parametric forms (e.g., Poisson, binomial)

4. There are no false positives, such as double counts

5. Sites are a random sample of the population

Generalized N-mixture model assumptions (Dail & Madsen 2011):

1. Sites and individual detections are independent
2. The abundance at site *i* at time *t* only depends on the abundance at site *i* at time *t-1* (i.e., Markov property)
3. All individuals present at site *i* at time *t* are assumed to have the same detection probability
4. Sites are a random sample of the population

S3.2: Mark-recapture model assumptions

Multistate Jolly-Seber model assumptions (Williams et al. 2002; Kéry & Schaub 2012):

1. Capture and survival rates are the same among individuals at a single capture occasion or any heterogeneity is modeled using covariates
2. Individuals behave independently with respect to capture and survival
3. Transitioning between states are a first-order Markov process. That is, a transition between states from time *t-1* to *t* depends only on the state at time *t*
4. The true state of each sampled individual is recorded without error during each capture event or any misclassifications are accounted for in the observation matrix
5. A single state transition occurs between time *t-1* and *t*
6. Capture occasions are instantaneous events
7. No animals are killed in the capture process

S4: Disentangling sampling and diagnostic errors

Abstract- Using infection intensity data, we describe a model to estimate pathogen prevalence and infection intensity of a host population during a single season while accounting for imperfect pathogen detection caused by field sampling and laboratory diagnostic failures. Typically, each individual is sampled and tested once. While straightforward, pathogens are routinely missed during sampling and diagnostic testing because of imperfect test sensitivity or

sampling protocol. These ‘false negatives’ result in an underestimate of pathogen prevalence and an overestimate of infection intensity. However, imperfect pathogen detection can easily be accounted for by formulating conditional probability statements when hosts are repeatedly sampled. Please note that we do not deal with threshold effects or detection limits of the diagnostic test, either specificity or sensitivity (e.g. if a qPCR reaction stops at 40 cycles rather than 50 cycles). Simulations of a pathogen-specific model than can account for threshold effects or detection limits, such as those outlined in Miller et al. 2012 or Lachish et al. 2012, may help with assessment. Here, we describe a model that takes into account sampling and diagnostic false negatives, and code to simulate and analyze data. The data input for this model are two 3-dimensional arrays: (1) the observations \(x_{i,j,k}\), denoting observed pathogen load from the sampling detection run *k* (e.g., qPCR) taken from sampling device *j* (e.g., swab or tissue sample) from host *i* and (2) the observations \(y_{i,j,k}\), denoting pathogen detection/non-detection from the sampling detection run *k* (e.g., qPCR) taken from sampling device *j* (e.g., swab or tissue sample) from host *i*

S4.1: Parameters of interest

List of state variables, observed data, and parameters of interest along with their definitions.

Quantity	Type	Definition
\(z_{i}\)	state variable	True infection state (*z* = 1 [infected] or 0 [uninfected]) of host *i*
\(N_{i}\)	state variable	True pathogen infection intensity of host *i*
\(w_{i,j}\)	state variable	True infection state of the sample *j* collected from host *i*
\(m_{i,j}\)	state variable	True pathogen infection intensity from sample *j* (e.g., swab or tissue sample) collected from host *i*
\(x_{i,j,k}\)	observed data	Observed pathogen load from sample *j* (e.g., swab or tissue sample) collected from host *i* during detection run *k* (e.g., qPCR)
\(y_{i,j,k}\)	observed data	Pathogen detection/non-detection from sample *j* (e.g., swab or tissue sample) collected from host *i* during detection run *k* (e.g., qPCR)
\(\psi\)	parameter	Pathogen prevalence of the host population

Quantity	Type	Definition
λ	parameter	Average host infection intensity
σ^2_{1}	parameter	Variance of infection intensities in the infected portion of the host population
$p_{1,i,j}$	parameter	Probability of detecting the pathogen on sampling device *j* (e.g., a swab or tissue sample), given that the host is infected
β_{0}	parameter	Intercept of the regression line between sampling detection probability, $p_{1,i,j}$, and true infection intensity N_{i} on the logit scale
β_{1}	parameter	Slope of the regression line between sampling detection probability, $p_{1,i,j}$, and true infection intensity N_{i} on the logit scale
σ^2_{2}	parameter	Measurement error in logged infection intensity from the sampling device *j* (e.g., a swab or tissue sample) for a given host
$p_{2,i,j}$	parameter	Probability of detecting the pathogen using a diagnostic method (e.g., qPCR), given that the sample device *j* collected from the host has the pathogen present
γ_{0}	parameter	Intercept of the regression line between the diagnostic test pathogen detection probability, $p_{2,i,j}$, and true infection intensity detected on the sampling device $m_{i,j}$
γ_{1}	parameter	Slope of the regression line between the diagnostic test pathogen detection probability, $p_{2,i,j}$, and true infection intensity detected on the sampling device $m_{i,j}$
σ^2_{3}	parameter	Measurement error in logged infection intensity from the diagnostic method (e.g., qPCR) for a given host

S4.2: Model outline

We model the true infection state z_{i} of host *i* as a Bernoulli random variable:

$$z_{i} \sim \textrm{Bernoulli}(\psi),$$

where ψ is the pathogen prevalence of the host population, $z_{i} = 1$ for infected hosts, and $z_{i} = 0$ for uninfected hosts.

Following Miller et al. (2012), we assume that the true infection intensity N_{i} for host *i* is lognormally distributed:

$$N_{i} \sim \textrm{lognormal}(\log(\lambda z_{i} + 0.001), \sigma^2_{1}),$$

where λ is the average infection intensity of infected hosts and σ^2_{1} represents the variation in host infection intensities. We add the small offset of 0.001 to λz_{i} because $\log(0)$ is undefined for uninfected hosts.

To account for imperfect pathogen detection caused by the sampling process, we specify a latent state variable, $w_{i,j}$, representing the true infection state of the sample *j* collected from host *i* as:

$$w_{i,j} \sim \textrm{Bernoulli}(p_{1,i,j} z_{i}).$$

If host *i* is infected (z_{i} = 1), we model the pathogen detection probability $p_{1,i,j}$ as a function of true infection intensity of the host *i* (N_{i} modeled above):

$$\textrm{logit}(p_{1,i,j}) = \beta_{0} + \beta_{1}\log(N_{i}),$$

to account for variability in the detection process dependent on host infection intensity.

We assume that the true infection intensity $m_{i,j}$ for sample *j* taken from host *i* is also lognormally distributed:

$$m_{i,j} \sim \textrm{lognormal}(\log(N_{i}w_{i,j} + 0.001), \sigma^2_{2}),$$ where N_{i} is the true infection intensity for host *i* and σ^2_{2} represents the logged measurement error caused by the sampling process (e.g., swabbing, collecting blood). Again, we add the small offset of 0.001 to $N_{i} w_{i,j}$ because $\log(0)$ is undefined for uninfected samples.

We model observed pathogen detection/non-detection $y_{i,j,k}$ during detection run *k* (e.g., qPCR) from sample *j* (e.g., swab or tissue sample) collected from host *i* as a Bernoulli random variable:

$$y_{i,j,k} \sim \textrm{Bernoulli}(p_{2,i,j}w_{i,j}),$$

where $p_{2,i,j}$ is the diagnostic test detection probability, $y_{i,j,k} = 1$ when the pathogen was detection, and $y_{i,j,k} = 0$ when it was not. If sample *j* from host *i* is infected ($w_{i,j}$ = 1), we model pathogen detection probability $p_{2,i,j}$ as a function of true infection intensity of sample *j* from host *i* ($m_{i,j}$ modeled above):

$$\textrm{logit}(p_{2,i,j}) = \gamma_{0} + \gamma_{1} * \textrm{log}(m_{i,j}),$$

to account for variability in the qPCR detection process dependent on sample infection intensity.

Finally, to account for the measurement error caused by diagnostic testing in pathogen infection intensities, we model the pathogen infection intensity $x_{i,j,k}$ during detection run *k* (e.g., qPCR) from sample *j* (e.g., swab or tissue sample) collected from host *i* is lognormally distributed:

$$x_{i,j,k} \sim \textrm{lognormal}(\textrm{log}(m_{i,j}+0.001), \sigma^2_{3}),$$

where $m_{i,j}$ is the average infection intensity for sample *j* from host *i* and σ^2_{3} represents the logged measurement error from the diagnostic test.

Fitting the model.- We fit models using Bayesian methods and estimated the posterior distributions for all parameters and latent states using Markov chain Monte Carlo (MCMC) implemented in JAGS 4.0.0 with the “jagsUI” package (Kellner, 2015) in the R environment (R Core Team 2018). We used vague priors, such as a normal distribution with mean zero and standard deviation 31.62 (precision = 0.001) or a normal distribution with mean zero and standard deviation 1.64 (Lunn et al. 2012) for all parameters. Lunn et al. (2012) recommends using a normal distribution with mean zero and standard deviation 1.64 as priors for parameters on the logit scale to create a uniform distribution between 0 and 1 on the probability scale when the values are back transformed. If a larger standard deviation is used, then the resulting back transformation to the probability scale will correspond to high probabilities around the extremes, i.e., 0 and 1. We use hyperpriors to calculate standard deviation when necessary using a gamma distribution (shape = 0.001, scale = 0.001). Although we have specified priors with little information, it is important to evaluate the effects of prior choice by varying priors within specific systems. We computed three chains for each random variable with diffuse initial values. We assess convergence by visually inspecting traceplots and using the Rhat diagnostics of Gelman (Brooks & Gelman 1998).

S4.3: Simulating data

Our first step in simulating data is to define the survey conditions and parameter estimates. If you are trying to determine how many samples or diagnostic tests your study requires, make your best guess at parameter values and examine how the precision and accuracy of recovered parameter estimates varies with the number of samples collected and analyzed.

```
set.seed(4)

n.ind  <- 300     # Number of individuals sampled
n.sample <- 4     # Number of samples collected per individual
n.diagnostic <- 4 # Number of diagnostic runs per samples

psi    <- 0.6     # True pathogen prevalence
lambda <- 4       # True average pathogen infection intensity

beta_0 <- 0      # Intercept of sample model on logit scale
beta_1 <- 0.25   # Slope of sample model on logit scale

gamma_0 <- 0     # Intercept of diagnostic test model on logit scale
gamma_1 <- 0.25  # Slope of diagnostic test model on logit scale

sigma1  <- 4    # Variance in host population infection intensity
sigma2  <- 1    # Measurement error in sampling of infection intensity
sigma3  <- 1    # Measurement error in diagnostic test of infection inten
sity
```

Generate the true infection status, *z*, and infection intensity of infected hosts, *N* for all individuals sampled in the population.

```
# True infection status of each host i
z <- rbinom(n.ind, 1, prob = psi)
  # 1 = infected
  # 0 = uninfected

# True infection intensity of each host i
N <- rlnorm(n.ind, meanlog = lambda, sdlog = sigma1)

# Assign 0 to each host with no infection (i.e., z = 0)
N <- N * z
```

Examine the data generated. Notice that only for individuals with z = 1 is there an infection intensity, *N*, assigned.

```
##    z        N
## 1  1  1822.45
## 2  1     2.09
## 3  1 25775.33
## 4  1 13334.41
## 5  0     0.00
## 6  1   493.44
## 7  0     0.00
## 8  0     0.00
## 9  0     0.00
## 10 1  4437.06
```

Next, we build in imperfect detection of the samples and measurement error of infection intensity caused by sampling.

```
# Create empty matrix to hold data
m <- w <- array(NA, dim = c(n.ind, n.sample))

# Next, calculate pathogen detection probability on samples
# In this scenario, pathogen detection probability on samples is postivel
y correlated to
# true host infection intensity
p_sample <- plogis(beta_0 + beta_1 * log(N))

# Generate the data for pathogen detection on each sample and the infecti
on intensity on
# each sample
for(i in 1:n.ind){       # For each individual i
  for(j in 1:n.sample){  # For each sample j

    # Determine if the pathogen was detected on a sample or not
    w[i, j] <- rbinom(1, 1, prob = p_sample[i])

    # Determine what the infection intensity detected was
    m[i, j] <- rlnorm(1, meanlog = log(N[i]), sdlog = sigma2)

  }
}

# Assign 0 to individuals that did not have pathogen detected
m <- m * w
```

Examine the data generated. Notice that only for individuals with $w = 1$ is there an infection intensity, m, assigned.

```
# Display matrix w (presence/absence of pathogen on sample)
  # rows = individuals
  # columns = replicate samples collected
head(w, n = 10)
```

```
##       [,1] [,2] [,3] [,4]
##  [1,]    1    1    1    1
##  [2,]    1    1    1    0
##  [3,]    1    1    1    1
##  [4,]    1    1    1    1
##  [5,]    0    0    0    0
##  [6,]    0    1    0    1
##  [7,]    0    0    0    0
##  [8,]    0    0    0    0
##  [9,]    0    0    0    0
## [10,]    1    1    1    1
```

```
# Display matrix m (infection infensity of pathogen on sample)
  # rows = individuals
  # columns = replicate samples collected
head(round(m, dig = 2), n = 10)
```

```
##           [,1]     [,2]     [,3]      [,4]
##  [1,]  1486.14   876.00  3029.56    144.74
##  [2,]     6.80     0.79     0.89      0.00
##  [3,] 21121.79 12193.71 80816.37 105151.69
##  [4,] 22380.49  4044.13  9434.66   8713.77
##  [5,]     0.00     0.00     0.00      0.00
##  [6,]     0.00  1857.01     0.00     93.84
##  [7,]     0.00     0.00     0.00      0.00
##  [8,]     0.00     0.00     0.00      0.00
##  [9,]     0.00     0.00     0.00      0.00
## [10,]  3939.35   665.19  2577.60   1615.49
```

Lastly, we will build in imperfect detection of the diagnostic method and measurement error of infection intensity caused by diagnostic test.

```
# Create empty array to hold data
x <- y <- array(NA, dim = c(n.ind, n.sample, n.diagnostic))

# Next, calculate pathogen detection probability of each diagnostic test
 run
# In this scenario, pathogen detection probability of the diagnostic test
 is
# postively correlated to the infection intensity on the samples collecte
d
p_diagnostic <- plogis(gamma_0 + gamma_1 * log(m))

for(i in 1:n.ind){            # For each individual i
  for(j in 1:n.sample){       # For each sample j
    for(k in 1:n.diagnostic){ # For each diagnostic run k

      # Determine if the pathogen was detected on a diagnostic test run
      y[i, j, k] <- rbinom(1, 1, prob = p_diagnostic[i, j])

      # Determine what the infection intensity on each diagnostic test ru
n was
      x[i, j, k] <- rlnorm(1, meanlog = log(m[i, j]), sdlog = sigma3)

    }
  }
}

# Assign 0 to individuals that did not have pathogen detected
x <- x * y
```

Examine the data generated. Notice that only for individuals with x = 1 is there an infection intensity, y, assigned. Here, we examine the the data generated for individuals 1 to 5 (rows), the samples collected 1 to 4 (columns), and the diagnostic runs 1 to 4 (sheets).

```
y[1:5, 1:n.sample, 1:n.diagnostic]
```

```
## , , 1
##
##      [,1] [,2] [,3] [,4]
## [1,]    0    0    1    1
## [2,]    1    1    0    0
## [3,]    1    0    1    1
## [4,]    1    1    1    1
## [5,]    0    0    0    0
##
## , , 2
##
##      [,1] [,2] [,3] [,4]
## [1,]    1    1    1    1
## [2,]    1    1    1    0
## [3,]    1    1    0    1
## [4,]    1    1    1    1
## [5,]    0    0    0    0
##
## , , 3
##
##      [,1] [,2] [,3] [,4]
## [1,]    1    1    0    0
## [2,]    1    0    0    0
## [3,]    1    1    1    1
## [4,]    1    0    1    1
## [5,]    0    0    0    0
##
## , , 4
##
##      [,1] [,2] [,3] [,4]
## [1,]    1    1    1    1
## [2,]    1    0    1    0
## [3,]    1    1    1    1
## [4,]    1    1    1    1
## [5,]    0    0    0    0
```

```
round(x[1:5, 1:n.sample, 1:n.diagnostic], dig = 2)
```

```
## , , 1
##
##          [,1]    [,2]     [,3]      [,4]
## [1,]     0.00    0.00  3714.21    211.95
## [2,]     3.64    1.61     0.00      0.00
## [3,] 14245.14    0.00 61328.59 283043.51
## [4,] 31953.49 1676.77  8758.20   4218.28
## [5,]     0.00    0.00     0.00      0.00
##
## , , 2
##
##          [,1]    [,2]     [,3]      [,4]
## [1,]  3889.31  128.63  3592.22    292.36
## [2,]    11.74    0.26     2.07      0.00
## [3,] 17949.67 2357.69     0.00 134360.84
## [4,]  7566.43 5880.73 12126.07   7756.57
## [5,]     0.00    0.00     0.00      0.00
##
## , , 3
##
##          [,1]    [,2]     [,3]     [,4]
## [1,]  1989.28  812.99     0.00     0.00
## [2,]     9.02    0.00     0.00     0.00
## [3,] 19997.27 6675.99 43731.02 54591.65
## [4,] 31161.49    0.00  3930.60  5116.33
## [5,]     0.00    0.00     0.00     0.00
##
## , , 4
##
##          [,1]     [,2]     [,3]      [,4]
## [1,]   606.38  6405.63  2630.98    124.64
## [2,]    14.43     0.00     0.21      0.00
## [3,] 11289.46  7233.74 55487.04 289995.97
## [4,] 24405.68 12360.60 12442.39   7043.11
## [5,]     0.00     0.00     0.00      0.00
```

Assign an NA where individuals were not infected. This makes it so that uninfected individuals are not taken into account when estimating average infection intensity.

```
N[N == 0] <- NA    # True infection intensity on host
m[m == 0] <- NA    # True infection intensity on sample
x[x == 0] <- NA    # Observed infection intensity on each sample
```

S4.4: JAGS code

Here, we specify the model in the JAGS language. Notice the similarities between the model specification and how we simulated the data.

```
{
    sink("model_sampling.txt")
    cat("
model{

# Prior
beta_0 ~ dnorm(0, 0.368)
beta_1 ~ dnorm(0, 0.368)
gamma_0 ~ dnorm(0, 0.368)
gamma_1 ~ dnorm(0, 0.368)

sigma1 ~ dgamma(0.001, 0.001)   # Standard deviation
tau1 <- 1 / (sigma1 * sigma1)   # Precision = (1/(sd * sd))

sigma2 ~ dgamma(0.001, 0.001)   # Standard deviation
tau2 <- 1 / (sigma2 * sigma2)   # Precision = (1/(sd * sd))

sigma3 ~ dgamma(0.001, 0.001)   # Standard deviation
tau3 <- 1 / (sigma3 * sigma3)   # Precision = (1/(sd * sd))

psi ~ dunif(0, 1)
lambda ~ dunif(0, 20)

#-Ecological Likelihood

for(i in 1:n.ind){  # For each individual i

 z[i] ~ dbern(psi)
    # The true infection status, z, of the ith individual is a
    # Bernoulli random variable, with probability psi.

 N[i] ~ dlnorm(log(lambda * z[i] + 0.001), tau1)
  # Given infection (z = 1), the infection intensity of
  # individual i is a random draw from a lognormal distributon.
  # Notice here too we add the small offset 0.001 to
  # log(lambda) because log(0) is undefined.

#-Sample Likelihood
 for(j in 1:n.sample){  # For each sample j
```

```
  w[i, j] ~ dbern(p1.eff[i, j])
    # The probability of detecting the pathogen on the ith
    # individual and jth sample is a Bernoulli random variable
    # with probability p1.eff given that the ith individual
    # is infected (z = 1)

    p1.eff[i, j] <- p1[i, j] * z[i]
    logit(p1[i, j]) <- beta_0 + beta_1 * log(N[i]+0.001)

  m[i, j] ~ dlnorm(log(N[i] * w[i, j] + 0.001), tau2)
    # We model sample pathogen infection intensity as a function of
    # true infection intensity of the ith individual (Ni modeled above)

#-Diagnostic test Likelihood
  for(k in 1:n.diagnostic){  # For each k diagnostic run

    y[i, j, k] ~ dbern(p.eff[i, j, k])
      # To account for imperfect pathogen detection caused
      # by diagnostic tests, we model the diagnostic test
      # detection probability, p2,i,j,k, from the ith individual
      # on the jth swab on the kth qPCR run, given that the ith
      # individual and jth swab were infected (wi,j = 1),
      # as a Bernoulli random variable

    p.eff[i, j, k] <- p2[i, j, k] * w[i, j]
    logit(p2[i, j, k]) <- gamma_0 + gamma_1 * log(m[i, j]+0.001)
      # We model diagnostic pathogen infection intensity as
      # a function of true sample infection intensity (m[i,j])

    x[i, j, k] ~ dlnorm(log(m[i, j] * y[i, j, k] + 0.001), tau3)

    } # ks
  } # js
} # is

}
",
        fill = TRUE)
```

```
    sink()
}
```

S4.5: Analyzing the data

To analyze the simualted data, we must bundle the data, generate intial values for latent state variables and parameters, and specify MCMC settings.

```
# Bundle the data
win.data <- list(
  y = y,
  x = x,
  n.ind = dim(y)[1],
  n.sample = dim(y)[2],
  n.diagnostic = dim(y)[3]
)

# Provide initial values for latent state variables
# This helps the model initialize faster
zinit <- z
ninit <- N
winit <- w
minit <- m

# We provide inital values for all other parameters
inits <- function() {list(
  beta_0 = beta_0,
  beta_1 = beta_1,

  gamma_0  = gamma_0,
  gamma_1  = gamma_1,

  sigma1  = sigma1,
  sigma2  = sigma2,
  sigma3  = sigma3,

  psi = psi,
  lambda = lambda,

  z = zinit,
  w = winit,

  m = minit,
  N = ninit
)}

# Name the parameters to monitor
```

```
params <- c("beta_0","beta_1",
            "gamma_0", "gamma_1",
            "sigma1","sigma2","sigma3",
            "psi", "lambda")

### MCMC settings
#ni <- 70000   # Number of iterations
#na <- 10000   # Number of iterations to adapt
#nb <- 20000    # Burn-in to discard
#nt <- 10      # Thinning rate
#nc <- 3       # Number of chains

ni <- 7   # Number of iterations
na <- 1   # Number of iterations to adapt
nb <- 2    # Burn-in to discard
nt <- 1      # Thinning rate
nc <- 3       # Number of chains

#- Run the model
library(jagsUI)
out <- jags(win.data, inits, params,
            "model_sampling.txt",
            n.chains = nc,
            n.thin = nt,
            n.iter = ni,
            n.burnin = nb,
            n.adapt = na,
            parallel = TRUE)
```

```
##
## Processing function input.......
##
## Done.
##
## Beginning parallel processing using 3 cores. Console output will be su
ppressed.
##
## Parallel processing completed.
##
## Calculating statistics.......
##
## Done.
```

Examine the output values and the traceplots to check for covergence and well mixing of chains.

```
print(out)
```

```
## JAGS output for model 'model_sampling.txt', generated by jagsUI.
## Estimates based on 3 chains of 7 iterations,
## adaptation = 1 iterations (possibly insufficient),
## burn-in = 2 iterations and thin rate = 1,
## yielding 15 total samples from the joint posterior.
## MCMC ran in parallel for 0.041 minutes at time 2018-11-05 09:41:27.
##
##               mean     sd      2.5%       50%     97.5% overlap0     f
## beta_0       0.204  0.126    -0.006     0.215     0.386     TRUE 0.933
## beta_1       0.239  0.022     0.196     0.248     0.267    FALSE 1.000
## gamma_0     -0.052  0.072    -0.167    -0.034     0.057     TRUE 0.867
## gamma_1      0.261  0.019     0.225     0.260     0.288    FALSE 1.000
## sigma1       3.228  0.160     2.970     3.301     3.398    FALSE 1.000
## sigma2       0.939  0.028     0.897     0.951     0.973    FALSE 1.000
## sigma3       0.994  0.012     0.980     0.990     1.020    FALSE 1.000
## psi          0.579  0.033     0.524     0.578     0.623    FALSE 1.000
## lambda       4.085  0.253     3.650     4.124     4.433    FALSE 1.000
## deviance 22962.945 16.325 22935.330 22967.903 22981.613    FALSE 1.000
##           Rhat n.eff
## beta_0   0.957    15
## beta_1   1.315     8
## gamma_0  1.395     7
## gamma_1  1.231    10
## sigma1   0.967    15
## sigma2   1.583     6
## sigma3   1.101    14
## psi      1.191    10
## lambda   1.141    15
## deviance 2.242     4
##
## **WARNING** Rhat values indicate convergence failure.
## Rhat is the potential scale reduction factor (at convergence, Rhat=1).

## For each parameter, n.eff is a crude measure of effective sample size.

##
## overlap0 checks if 0 falls in the parameter's 95% credible interval.
## f is the proportion of the posterior with the same sign as the mean;
## i.e., our confidence that the parameter is positive or negative.
```

```
##
## DIC info: (pD = var(deviance)/2)
## pD = 59.1 and DIC = 23022.04
## DIC is an estimate of expected predictive error (lower is better).
```

```
plot(out)
```

Trace of beta_0

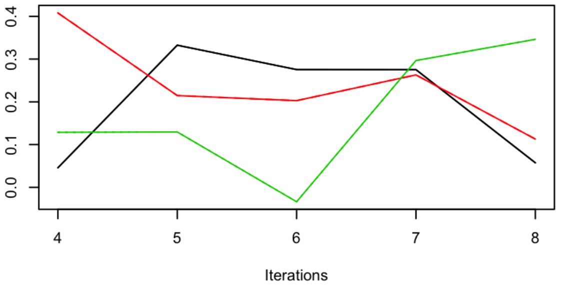

Density of beta_0

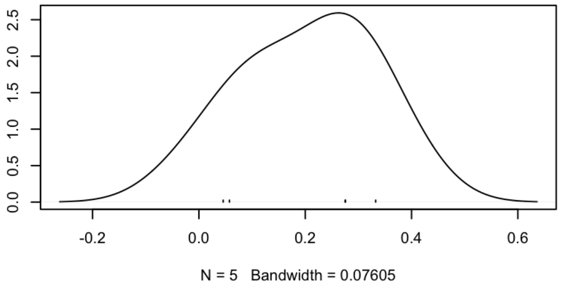

Trace of beta_1

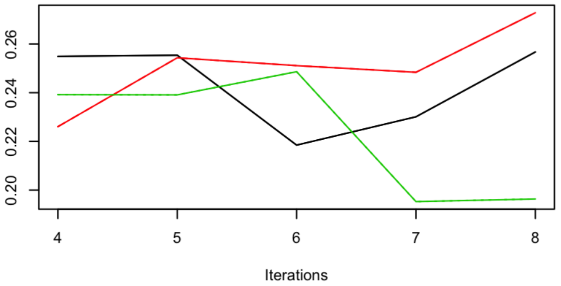

Density of beta_1

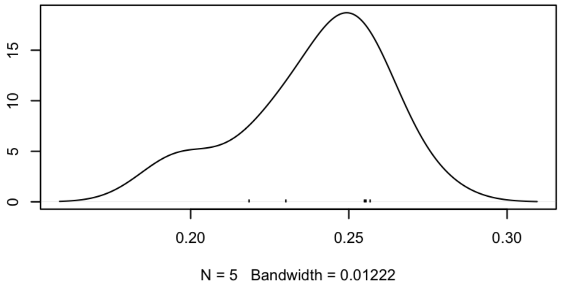

Trace of gamma_0

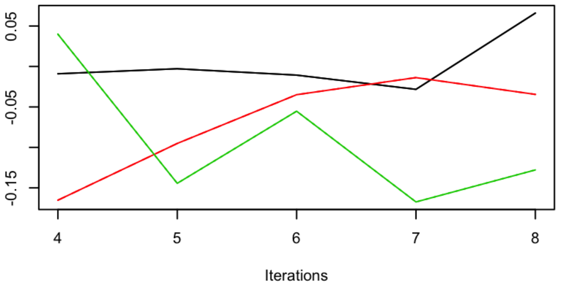

Density of gamma_0

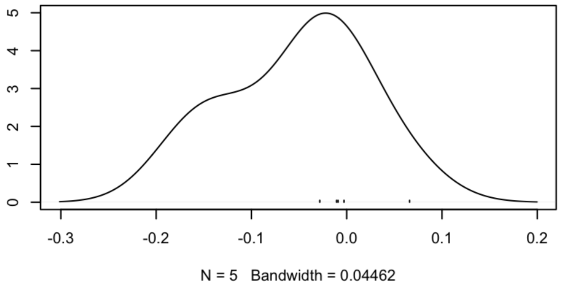

Trace of gamma_1

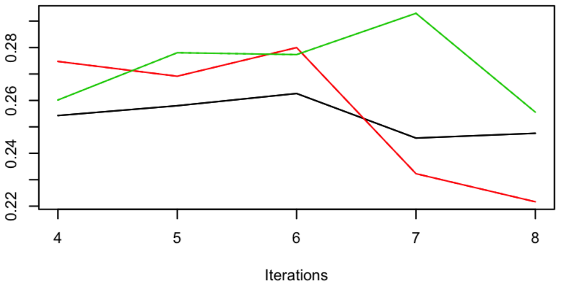

Density of gamma_1

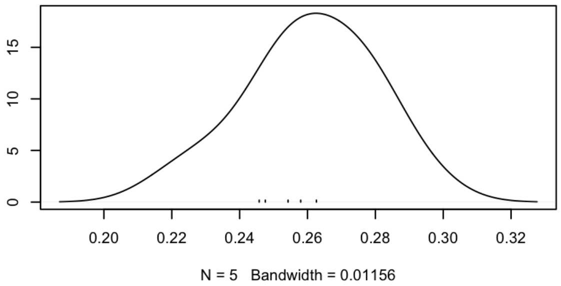

Trace of sigma1

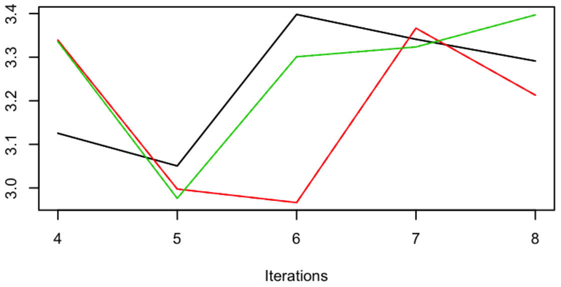

Density of sigma1

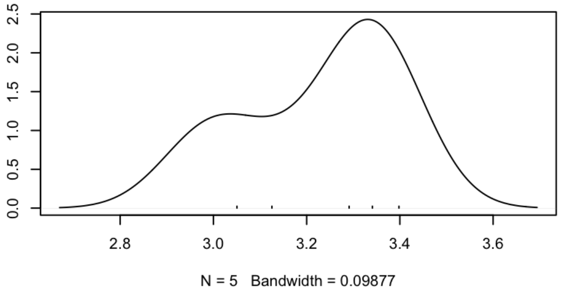

Trace of sigma2

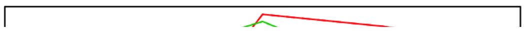

Density of sigma2

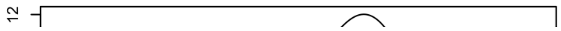

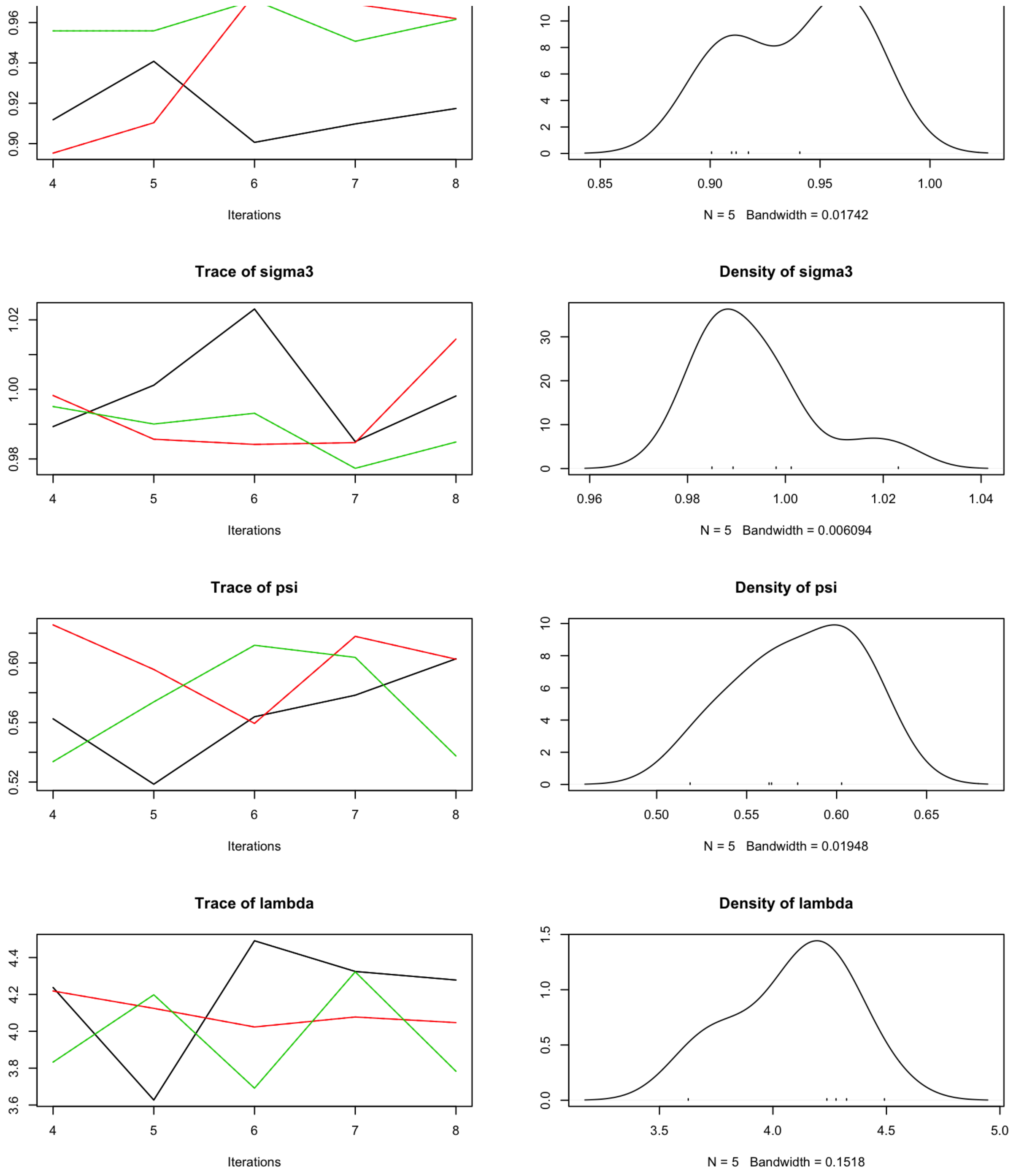
Iterations
N = 5 Bandwidth = 0.01742
Trace of sigma3
Density of sigma3
Iterations
N = 5 Bandwidth = 0.006094
Trace of psi
Density of psi
Iterations
N = 5 Bandwidth = 0.01948
Trace of lambda
Density of lambda
Iterations
N = 5 Bandwidth = 0.1518

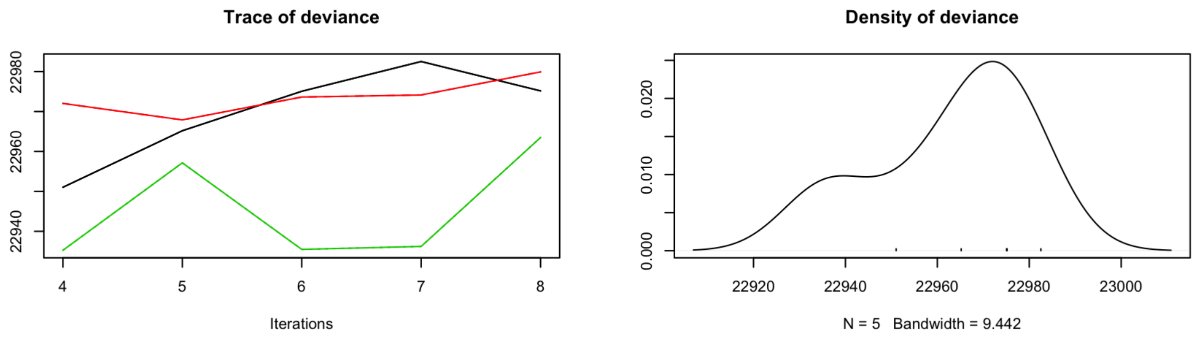

Next, we compare the true parameter estimates to the model estimation.

```
# Store the true paramter values
true <- c(beta_0,beta_1,
          gamma_0, gamma_1,
          sigma1,sigma2,sigma3,
          psi, lambda)

# Store the parameter names
names <- c("beta_0","beta_1",
            "gamma_0", "gamma_1",
            "sigma1","sigma2","sigma3",
            "psi", "lambda")

# Extract the model mean
mod.mean <- c(
  out$mean$beta_0,
  out$mean$beta_1,
  out$mean$gamma_0,
  out$mean$gamma_1,
  out$mean$sigma1,
  out$mean$sigma2,
  out$mean$sigma3,
  out$mean$psi,
  out$mean$lambda)

# Extract the lower credible interval
mod.q2.5 <- c(
  out$q2.5$beta_0,
  out$q2.5$beta_1,
  out$q2.5$gamma_0,
  out$q2.5$gamma_1,
  out$q2.5$sigma1,
  out$q2.5$sigma2,
  out$q2.5$sigma3,
  out$q2.5$psi,
  out$q2.5$lambda)

# Extract the upper credible interval
mod.q97.5 <- c(
  out$q97.5$beta_0,
```

```
  out$q97.5$beta_1,
  out$q97.5$gamma_0,
  out$q97.5$gamma_1,
  out$q97.5$sigma1,
  out$q97.5$sigma2,
  out$q97.5$sigma3,
  out$q97.5$psi,
  out$q97.5$lambda)

# Combine names, truth, mean, lower CI, and upper CI
dat <- data.frame(names = names,
                  true = true,
                  mod.mean = mod.mean,
                  mod.q2.5 = mod.q2.5,
                  mod.q97.5 = mod.q97.5)

# Load library
library(ggplot2)

# Indicate colors for the plot
cols <- c("Truth" = "red", "Estimated" = "black")

# Plot
ggplot(dat, aes(x= names, y=mod.mean))+
  geom_linerange(size = 1, aes(ymin=mod.q2.5, ymax=mod.q97.5)) +
  geom_point(size = 3, aes(x = names, y = mod.mean, col = "Estimated")) +
  geom_point(size = 3, aes(x = names, y = true, col = "Truth")) +
  scale_colour_manual("Values", values=cols)+
  geom_hline(yintercept = 0, lty=2) +
  coord_flip() + ylab('Parameter estimates') +
  xlab("Parameter names") +
  theme_bw()+
  theme(axis.text.x = element_text(size = 17, color = "black"),
        axis.text.y = element_text(size = 17, color = "black"),
        axis.title.y = element_text(size = 17, color = "black"),
        axis.title.x =element_text(size = 17, color = "black"),
        legend.title =element_text(size = 17, color = "black"),
        legend.text =element_text(size = 17, color = "black"),
```

```
        panel.grid.major = element_blank(),
        panel.grid.minor = element_blank())
```

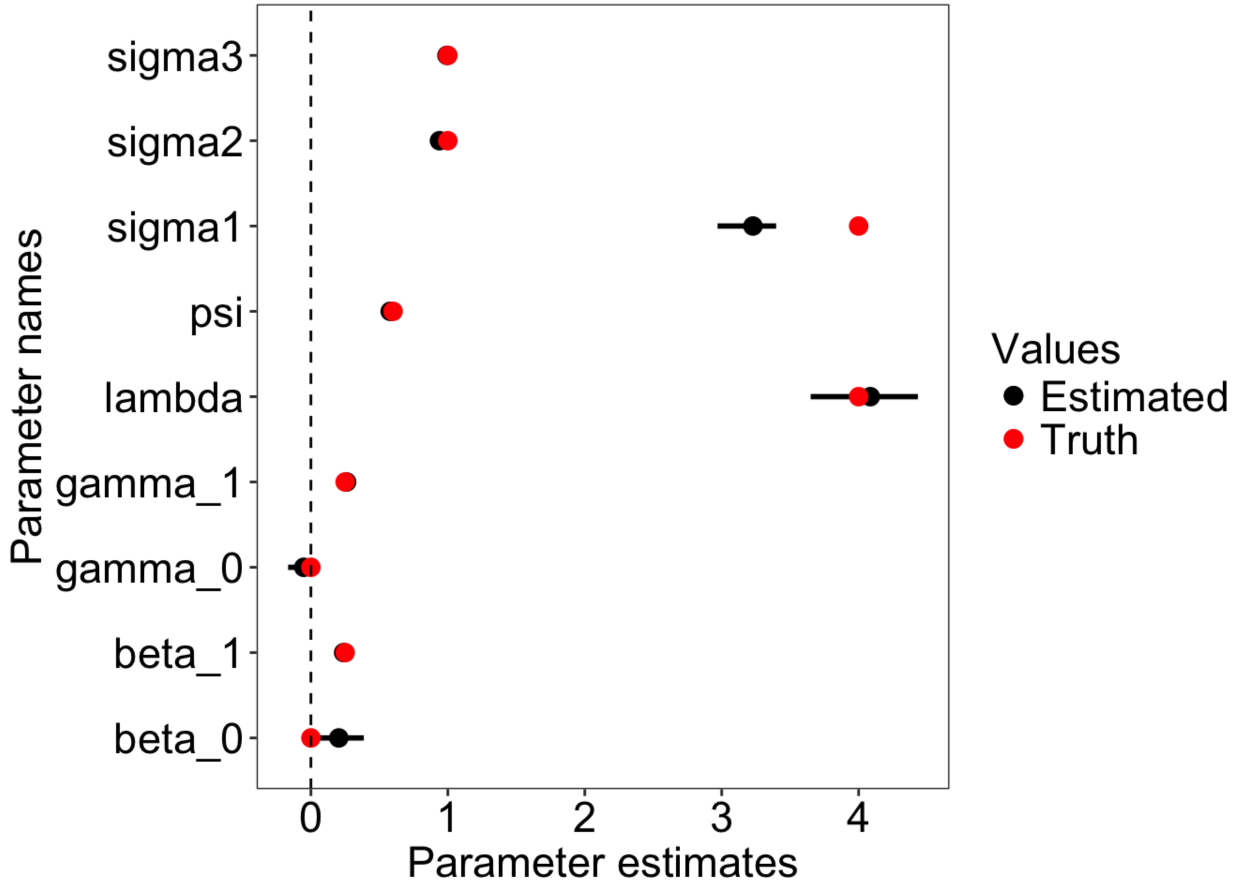

S5: Disease-structured N-mixture model (single season)

Abstract- In this section, we introduce the single season disease-structured *N*-mixture model that accounts for imperfect host and pathogen detection. We provide a list of state variables, parameters of interest, and the model outline. We highly encourage anyone interested in using this model for their own work or planning a study to use this code as a template for a power analysis. This formulation of the model assumes that there are no informative priors for pathogen detection probability. Therefore, the data collected are: (1) repeated temporal visits to several sites a season and (2) collecting multiple samples from a single host seen at each site during each visit.

S5.1: Table

List of state variables, observed data, and parameters of interest along with their definitions.

Quantity	Type	Definition
$N_{N,i}$	state variable	True abundance of uninfected hosts at site i
$N_{I,i}$	state variable	True abundance of infected hosts at site i
$E_{i,j}$	state variable	True average infection intensity at site i during survey j
$Z_{i,j,k}$	state variable	True average pathogen infection intensity at site i during survey j on host k
$m_{i,j}$	state variable	Number of individuals that were incorrectly classified as “uninfected” when really “infected” at site i during survey j
$y_{i,j,k,l}$	state variable	Presence/absence of the pathogen at site i during survey j on host k on sample l
ψ_{i}	state variable	True presence of an infected host at site i
$I_{i,j,k,l}$	observed data	Pathogen infection infensity at site i during survey j on host k on sample l
$w_{i,j,k,l}$	observed data	Detection/non-detection of the pathogen at site i during survey j on host k on sample l
$\alpha.lamI$	parameter	Average abundance of infected hosts at a site
$\alpha.lamN$	parameter	Average abundance of uninfected hosts at a site
p_I	parameter	Probability of detecting an infected host
p_N	parameter	Probability of detecting an uninfected host
μ_{E}	parameter	True average infection intensity across all sites, surveys, hosts, and samples
$sd.E$	parameter	Variance in infection intensities of the infected portion of the host population

Quantity	Type	Definition
$sd.Z$	parameter	Measurement error in logged infection intensity from a host and sampling device (e.g., a swab or tissue sample)
$sd.I$	parameter	Measurement error in logged infection intensity from a sampling device (e.g., a swab or tissue sample)
$\delta0$	parameter	Intercept of the regression line between the sampling detection probability, p_{I}, and true infection intensity $E_{i,j}$ on the logit scale
$\delta1$	parameter	Slope of the regression line between the sampling detection probability, p_{I}, and true infection intensity $E_{i,j}$ on the logit scale

S5.2: Model outline

Pathogen model

We specify the observation model to account for imperfect host and pathogen detection during the sampling process by assuming that pathogen detection is related to infection intensity (e.g., Lachish et al. 2012; Miller et al. 2012; DiRenzo et al. 2018). We denote $g_{s,i,j}$ as the number of hosts detected in each disease state *s* (where *s*=1 for uninfected and *s*=2 for infected) at site *i* during survey *j*. We assume that there are a number of misidentified infected hosts, $m_{i,j}$ (i.e., the number of hosts classified as uninfected when they are actually infected). The corrected number of detected uninfected and infected hosts, $y_{s,i,j}$, in disease state *s* at site *i* during survey *j* is part of a deterministic relationship between the number of observed hosts, $g_{s,i,j}$, and the number of misspecified hosts, $m_{i,j}$:

$$g_{1,i,j} = y_{1,i,j} + m_{i,j},$$ $$g_{2,i,j} = y_{2,i,j} - m_{i,j}.$$

We assume that the number of misspecified individuals, $m_{i,j}$, is a binomial random variable dependent on the average pathogen detection probability for hosts at site *i* during survey replicate *j*, $\theta_{i,j}$: $$m_{i,j} \sim \textrm{binomial}(y_{2,i,j}, 1 - \theta_{i,j}).$$ To estimate $\theta_{i,j}$, we model the relationship between pathogen detection probability and average pathogen infection intensity, $E_{i,j}$: logit($\theta_{i,j}$) = $\delta0$ + $\delta1$ log($E_{i,j}$+1), where $\delta0$ and $\delta1$ are the intercept and slope parameters determined below.

We calculate $\delta0$ and $\delta1$ by first modeling the average pathogen infection intensity at site i during survey j, $E_{i, j}$, as lognormally distributed with log mean, μ_{E}, and log standard deviation, $sd.E$, such that: $$E_{i, j} \sim \textrm{lognormal}(log(\mu_{E}+0.001), sd.E)$$ Next, we estimate the average pathogen infection intensity at site i during survey j for each host k, $Z_{i, j,k}$, as lognormally distributed with log mean, $E_{i, j}$, and log standard deviation, $sd.Z$, such that: $$Z_{i, j,k} \sim \textrm{lognormal}(log(E_{i, j}+0.001), sd.Z)$$ Using the observed data collected, the pathogen infection infensity at site i during survey j for each host k on sample l, $I_{i,j,k,l}$, we assume that it also is lognormally distributed with log mean, $Z_{i, j,k}$, and log standard deviation, $sd.I$: $$I_{i,j,k,l} \sim \textrm{lognormal}(log(Z_{i,j,k}+0.001), sd.I)$$ Again, using the data collected, we determine if site i during survey j for host k on sample l is infected, $w_{i, j, k, l}$, where $w = 1$ represents the site has the pathogen present and $w = 0$ represents the absence of the pathogen, using the information from the host model, such that ψ_{i} equal 1 when an infected host is present and ψ_{i} equal 0 when infected hosts are absent, multiplied by pathogen detection probability, $\eta_{i,j,k,l}$: $$w_{i, j, k, l}\sim \textrm{Bernoulli}(\psi \eta_{i,j,k,l}).$$ Then, we specify the relationship between $\eta_{i,j,k,l}$ and $I_{i,j,k,l}$ using: $$logit(\eta_{i,j,k,l}) = \delta0 + \delta1 log(I_{i,j,k,l}+0.001).$$

Host model

We incorporate imperfect host detection during the sampling process by modeling the true number of hosts $N_{s,i,j}$ in disease class s at site i captured in survey j as a binomial random variable: $$y_{s,i,j} \sim \textrm{binomial}(N_{s,i}, p_s),$$ whose parameters are the host detection probability p_{s} (which we allow to differ between disease states) and the true host abundance $N_{s,i}$ in disease class s at site i. We model true host abundance $N_{s,i}$ in disease class s and site i as a Poisson random variable, where μ_{s} (1 = uninfected and 2 = uninfected) is the mean host abundance of disease state s across all sites:

$$N_{s,i} \sim \textrm{Poisson}(\mu_{s}).$$

We compute total host abundance T_{i} and pathogen prevalence D_{i} as derived quantities from the host model parameters. Total host abundance T_{i} at site i is calculated as:

$$\tilde{N}=N_{1,i}+N_{2,i},$$

and pathogen prevalence P_{i} at site i is computed as:

$$P_{i}=\frac{N_{2,i}}{\tilde{N}}.$$

S5.3: Simulating data

Write a function to simulate the data.

```
R = 100 # Number of sites
T = 3   # Number of replicate surveys per site
L = 3   # Number of replicate samples collected per individual
alpha.lamN = 5   # Average number of uninfected hosts per site the first
 season
alpha.lamI = 4   # Average number of infected hosts per site the first se
ason
pN = 0.8  # Detection probability of uninfected hosts during each survey
pI = 0.7  # Detection probability of infected hosts during each survey
delta0 = -0.25  # Imperfect pathogen detection intercept (logit scale)
delta1 = 0.5    # Imperfect pathogen detection slope (logit scale)
sd.Z = 1  # Standard deviation
sd.I = 1  # Standard deviation
sd.E = 1  # Standard deviation
mu.E = 3  # Average site-level pathogen infection intensity

  #-------- Average true host abundance
  NN <- rpois(n = R, lambda = alpha.lamN)
  NI <- rpois(n = R, lambda = alpha.lamI)



  # Host obervation process
  yN <- yI <- array(NA, dim = c(R, T))
  for(i in 1:R){

    for(j in 1:T){


        yN[i, j] <- rbinom(n = 1, NN[i], pN)
        yI[i, j] <- rbinom(n = 1, NI[i], pI)


      }
    }

  # Which sites have infected hosts?
  psi <- as.numeric(NI>0)

  # Pathogen observation process
  K <- yI
  I <- theta <- w <- array(NA, dim = c(R, T, max(K), L))
```

```
  m <- array(NA, dim = c(R, T))
  Z <- array(NA, dim = c(R, T, max(K)))
  E <- eta <- m <- array(NA, dim = c(R, T))

  for(i in 1:R){  # For each site

    for(j in 1:T){ # For each survey

      # Average site-pathogen infection intensity
        E[i, j] <- rlnorm(1, meanlog =  mu.E, sdlog = sd.E)

      if(yI[i, j] > 0){

        for(k in 1:K[i,j]){ # For each individual

          # Average site-pathogen infection intensity across hosts
            Z[i, j, k] <- rlnorm(1, meanlog =  log(E[i, j]+ 0.001), sdlog
 = sd.Z)

          for(l in 1:L){ # For each sample

            #Observed Average site-pathogen infection intensity across ho
sts and samples
            I[i, j, k, l] <- rlnorm(1, meanlog =  log(Z[i, j, k]+ 0.001),
 sdlog = sd.I)

          # If there are infected individuals- assign the average site-le
vel infection intensity during each survey from each hosts and their mult
iple samples
            theta[i,j,k,l] <- plogis(delta0 + delta1 * log(I[i,j,k,l]+ 0.
001))

            w[i, j, k, l] <- rbinom(1, 1, p = (theta[i,j,k,l] * psi[i]))

          }
        }
      }
    }
  }
```

```
# Misspecified hosts

for(i in 1:R){
  for(j in 1:T){

    eta[i,j] <- plogis(delta0 + delta1 * log(E[i,j]+ 0.001))
    # The individuals that would be misidentified are the uninfected ones
 that should be infected
    m[i,j] <- rbinom(1, yI[i,j], p = (1-eta[i,j]))

  }
}

# the observed uninfected data = corrected # of hosts that are uninfected
 - # that are really infected
gN = yN + m

# the observed infected data = corrected # of hosts that are infected + #
 that are really infected
gI = yI - m
```

S5.4: JAGS code

```
{
sink("model.txt")
cat("
model{

# Priors

#------- NOT Infected
alpha.lamN  ~ dnorm(0,0.01) # Average abundance of uninfected hosts the f
irst season
pN     ~ dunif(0,1) # Detection probability of uninfected hosts

#------- Infected
alpha.lamI  ~ dnorm(0,0.01)# Average abundance of infecteds the first sea
son
pI     ~ dunif(0,1) # Detection probability of infected

#------------ Ecological model

for(i in 1:R){
#------ Not infected
  NN[i] ~ dpois(alpha.lamN)

#----- Infected
  NI[i] ~ dpois(alpha.lamI)
}

#-------Host Obervation model

for(i in 1:R){
  for(j in 1:T){


    yN[i, j] ~ dbin(pN, NN[i])
    yI[i, j] ~ dbin(pI, NI[i])


  }
}

#---- Infection intensity
```

```
tau.I <- 1/(sd.I * sd.I)
sd.I ~ dgamma(0.01, 0.01)

tau.Z <- 1/(sd.Z *sd.Z)
sd.Z ~ dgamma(0.01, 0.01)

mu.E ~ dnorm(0, 0.01)
tau.E <- 1/(sd.E * sd.E)
sd.E ~ dgamma(0.01, 0.01)

delta0 ~ dnorm(0, 0.368)
delta1 ~ dnorm(0, 0.368)

# equals function is logical (TRUE/FALSE)

psi[1:R] <- (1 - equals(NI[1:R], 0))
    # psi = 0 then 0 infected hosts
    # psi = 1, then > 0 infected hosts



for(i in 1:R){   # For each site

  for(j in 1:T){  # For each survey
          # True average infection intensity across sites and surveys
           E[i, j] ~ dlnorm(mu.E, tau.E)

    for(k in 1:K[i,j]){ # For each host
          # True average infection intenstiy across sites, surveys, and h
osts
            Z[i, j, k] ~ dlnorm(log(E[i, j]+ 0.001), tau.Z)

        for(l in 1:L){  # For each sample
          # Observed infection intensity
            I[i, j, k, l] ~ dlnorm(log(Z[i, j, k]+ 0.001), tau.I)

          # probability of infection
           w[i, j, k, l] ~ dbern(p.eff[i, j, k, l])

            p.eff[i, j, k, l] <- theta[i, j, k, l] * psi[i]
```

```
          # theta = pathogen detection probability
          # The relationship between pathogen detection probability and i
nfection intensity
            logit(theta[i, j, k, l]) <- delta0 + delta1 * log(I[i, j, k,
 l]+.001)

      }
    }
   }
 }

#----- Correcting for misspecified hosts

D0 <- delta0
D1 <- delta1

for(i in 1:R){    # For each site
 for(j in 1:T){  # For each survey

 # Add/subtract the number of individuals that were misspecified as uninf
ected, when #truth was they were infected
   gI[i, j] ~ dsum(yI[i, j], (-1 * m[i, j]))
   gN[i, j] ~ dsum(yN[i, j], m[i, j])

 # Calculate the number of misspecified hosts
   m[i, j] ~ dbin(eta1[i, j], yI[i, j])
   eta1[i, j] <- 1 - eta[i, j]
   logit(eta[i, j]) <- D0 + D1 * log(E[i, j]+ 0.001)
 }

}


}
", fill = TRUE)
sink()
}
```

S5.5: Analyzing the data

Similar as before, we simulate the data, bundle the data, create initial values, list the parameters to monitor, set the MCMC settings, and run the model.

```
# Bundle data
win.data <- list(gN = gN,
                 gI = gI,
                 I = I,
                 w = w,

                 K = K,
                 R = dim(yN)[1],
                 T = dim(yN)[2],
                 L = dim(I)[4])

# Initial values
inits <- function() {list(
  alpha.lamN = alpha.lamN,
  pN = pN,

  alpha.lamI = alpha.lamI,
  pI = pI,

  mu.E = mu.E,
  sd.E = sd.E,
  sd.I = sd.I,
  sd.Z = sd.Z,
  delta0= delta0,
  delta1= delta1,

  m =  m,
  NN = NN,
  NI = NI,

  yI = yI,
  yN = yN,

  E = E,
  Z = Z

)}

# Monitor Parameters
```

```
params <- c("alpha.lamN", "alpha.lamI",
            "pN", "pI",
            "mu.E",
            "sd.E",
            "sd.I",
            "sd.Z",
            "delta0", "delta1"
)
#
## MCMC settings
#ni <- 10000   # Number of iterations
#na <- 10000   # Number of iterations to adapt
#nb <- 1000   # Burn-in to discard
#nt <- 10      # Thinning rate
#nc <- 3       # Number of chains
ni <- 7   # Number of iterations
na <- 1   # Number of iterations to adapt
nb <- 2    # Burn-in to discard
nt <- 1      # Thinning rate
nc <- 3       # Number of chains
library("jagsUI")

out <- jags(win.data, inits, params, "model.txt",
            n.chains = nc, n.thin = nt, n.iter = ni, n.adapt = na,
            n.burnin = nb, parallel = TRUE)
```

```
##
## Processing function input.......
##
## Done.
##
## Beginning parallel processing using 3 cores. Console output will be su
ppressed.
##
## Parallel processing completed.
##
## Calculating statistics.......
##
## Done.
```

Examine the output values and the traceplots to check for covergence and well mixing of chains.

```
print(out)
```

```
## JAGS output for model 'model.txt', generated by jagsUI.
## Estimates based on 3 chains of 7 iterations,
## adaptation = 1 iterations (possibly insufficient),
## burn-in = 2 iterations and thin rate = 1,
## yielding 15 total samples from the joint posterior.
## MCMC ran in parallel for 0.086 minutes at time 2018-11-05 09:41:31.
##
##                  mean     sd      2.5%       50%     97.5% overlap0 f
Rhat
## alpha.lamN     5.359  0.212     5.056     5.343     5.690    FALSE 1
1.325
## alpha.lamI     4.105  0.180     3.849     4.077     4.354    FALSE 1
1.345
## pN             0.794  0.011     0.770     0.796     0.809    FALSE 1
1.254
## pI             0.719  0.012     0.703     0.715     0.739    FALSE 1
1.105
## mu.E           3.042  0.063     2.934     3.038     3.152    FALSE 1
1.043
## sd.E           0.965  0.040     0.915     0.954     1.027    FALSE 1
1.831
## sd.I           1.001  0.012     0.980     0.999     1.019    FALSE 1
1.848
## sd.Z           1.019  0.024     0.985     1.012     1.054    FALSE 1
1.040
## delta0        -0.206  0.063    -0.300    -0.190    -0.111    FALSE 1
1.244
## delta1         0.515  0.024     0.479     0.517     0.547    FALSE 1
1.271
## deviance   26729.146 32.348 26682.288 26718.643 26770.797    FALSE 1
1.829
##            n.eff
## alpha.lamN     8
## alpha.lamI     7
## pN            10
## pI            13
## mu.E          15
## sd.E           5
## sd.I           5
```

```
## sd.Z           15
## delta0          9
## delta1          8
## deviance        5
##
## **WARNING** Rhat values indicate convergence failure.
## Rhat is the potential scale reduction factor (at convergence, Rhat=1).

## For each parameter, n.eff is a crude measure of effective sample size.


##
## overlap0 checks if 0 falls in the parameter's 95% credible interval.
## f is the proportion of the posterior with the same sign as the mean;
## i.e., our confidence that the parameter is positive or negative.
##
## DIC info: (pD = var(deviance)/2)
## pD = 296.8 and DIC = 27025.95
## DIC is an estimate of expected predictive error (lower is better).
```

```
plot(out)
```

Trace of alpha.lamN

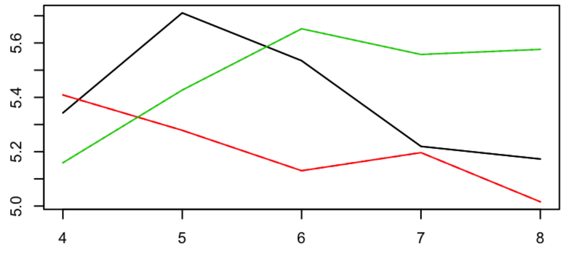

Density of alpha.lamN

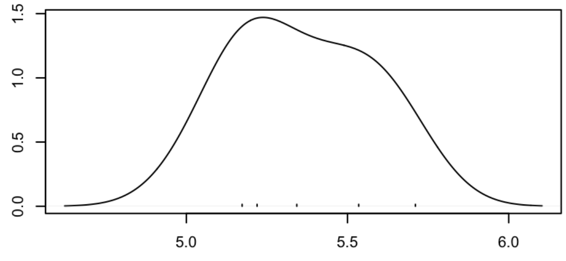

Trace of alpha.lamI

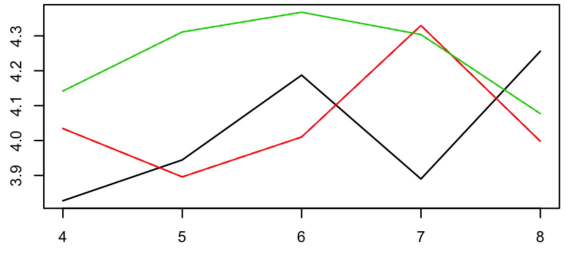

Density of alpha.lamI

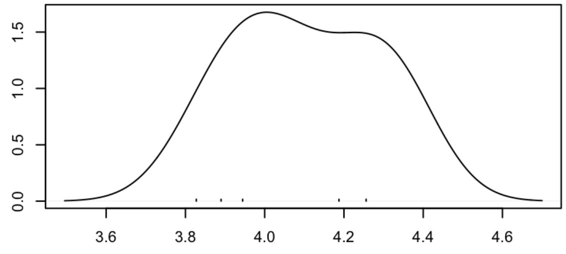

Trace of pN

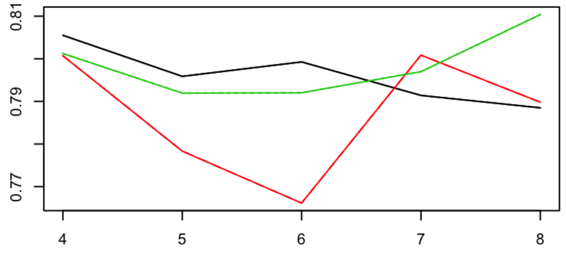

Density of pN

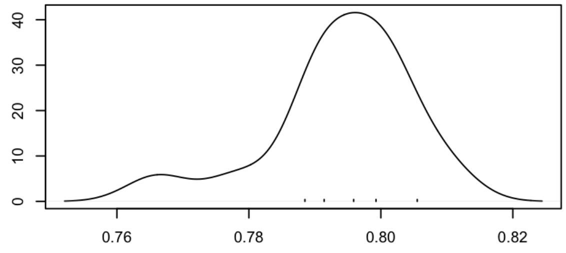

Trace of pI

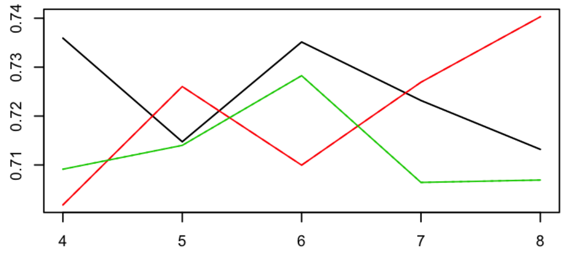

Density of pI

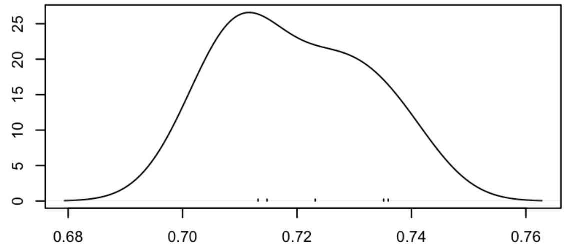

Trace of mu.E

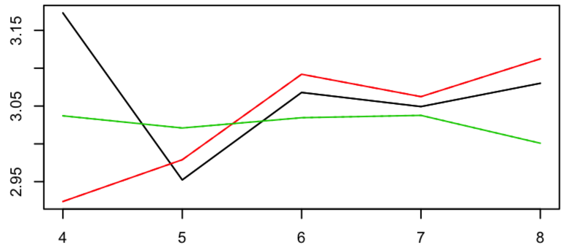

Density of mu.E

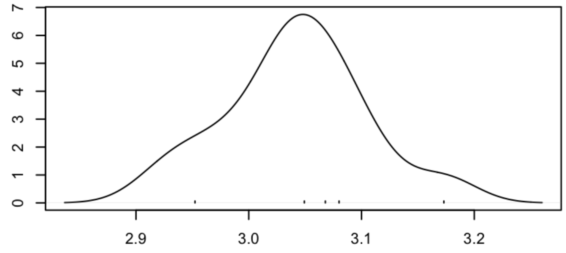

Trace of sd.E

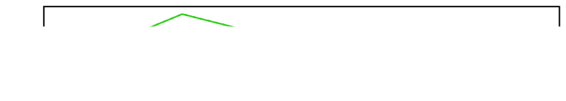

Density of sd.E

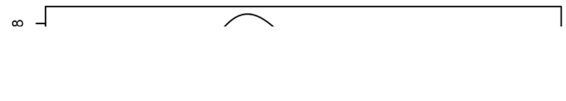

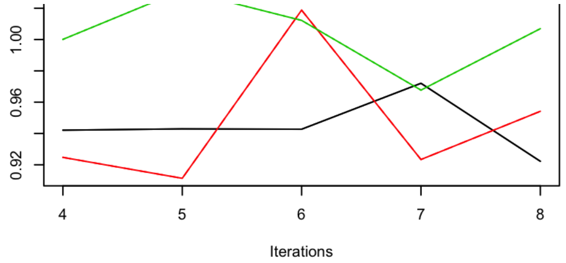

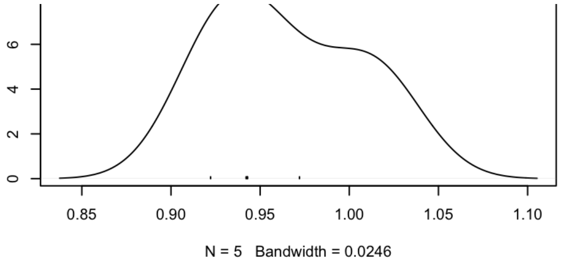

Trace of sd.I

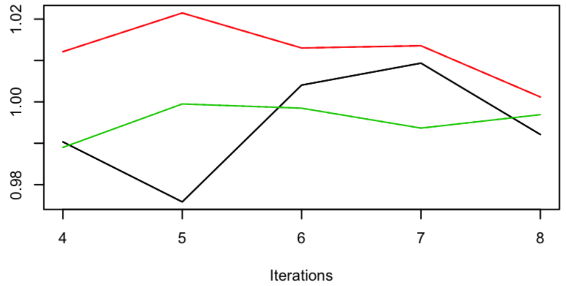

Density of sd.I

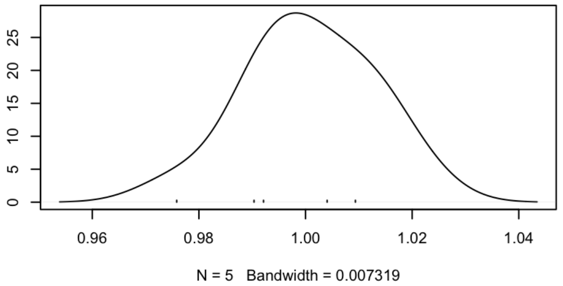

Trace of sd.Z

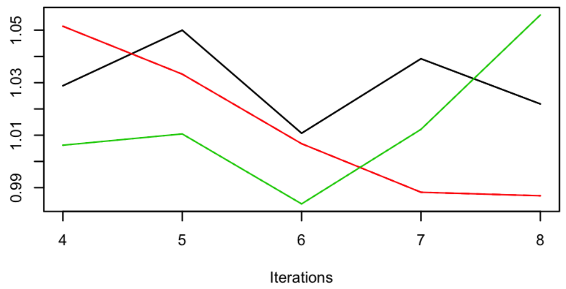

Density of sd.Z

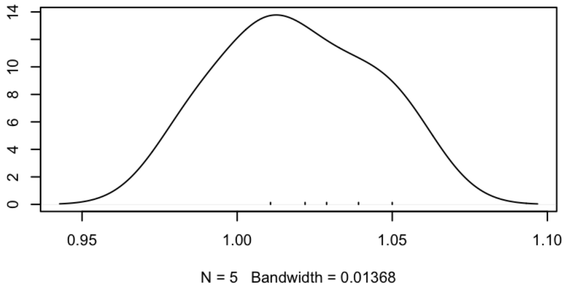

Trace of delta0

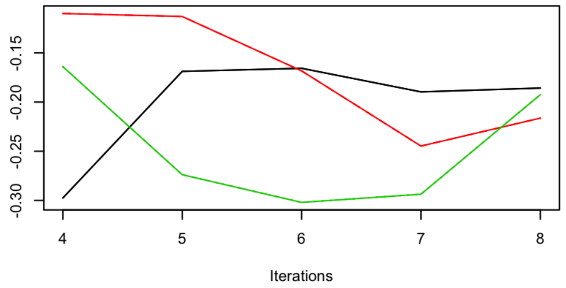

Density of delta0

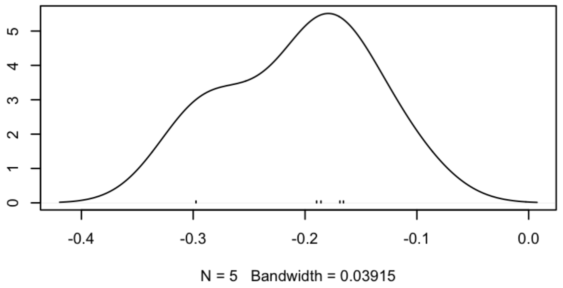

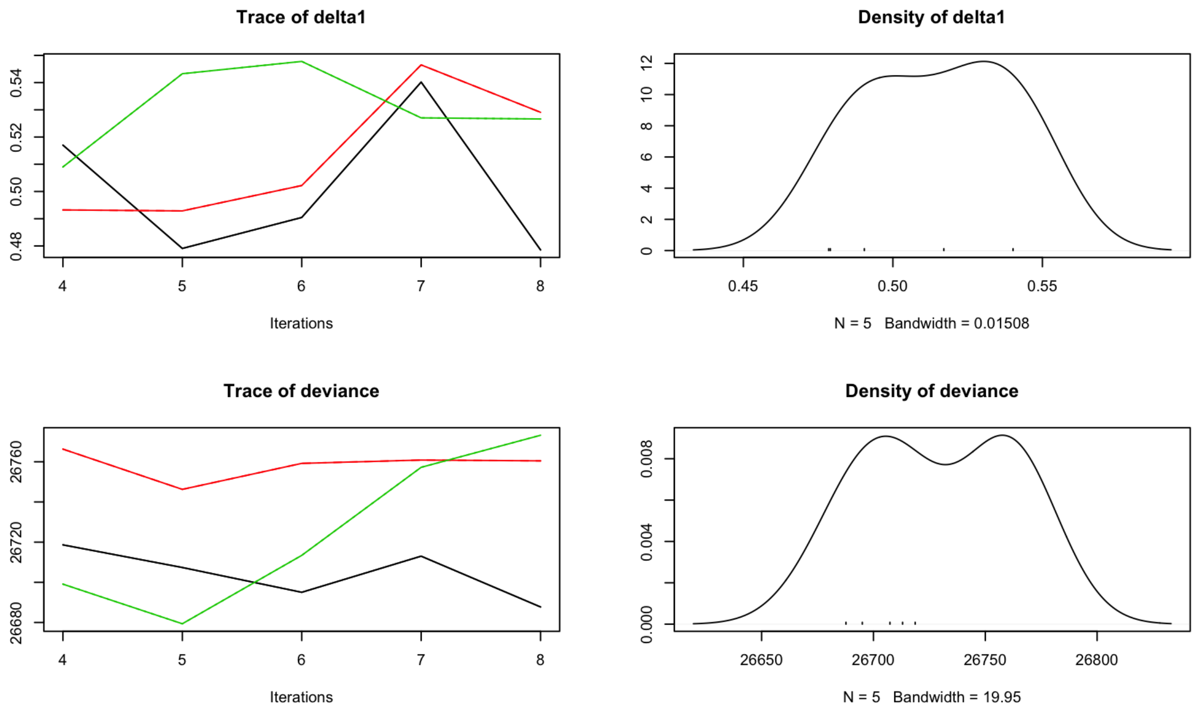

Next, we compare the true parameter estimates to the model estimation.

```
# Store the true paramter values
true <- c((alpha.lamN),
          (alpha.lamI),
          (pN),
          (pI),
          (mu.E),
          (delta0),
          (delta1)
)

# Store the parameter names
names <- c("alpha.lamN", "alpha.lamI",
           "pN", "pI",
           "mu.E",
           "delta0", "delta1"
)

# Extract the model mean
mod.mean <- c(
  out$mean$alpha.lamN,
  out$mean$alpha.lamI,
  out$mean$pN,
  out$mean$pI,
  out$mean$mu.E,
  out$mean$delta0,
  out$mean$delta1)

# Extract the lower credible interval
mod.q2.5 <- c(
  out$q2.5$alpha.lamN,
  out$q2.5$alpha.lamI,
  out$q2.5$pN,
  out$q2.5$pI,
  out$q2.5$mu.E,
  out$q2.5$delta0,
  out$q2.5$delta1)

# Extract the upper credible interval
mod.q97.5 <- c(
```

```
  out$q97.5$alpha.lamN,
  out$q97.5$alpha.lamI,
  out$q97.5$pN,
  out$q97.5$pI,
  out$q97.5$mu.E,
  out$q97.5$delta0,
  out$q97.5$delta1)

# Combine names, truth, mean, lower CI, and upper CI
dat <- data.frame(names = names,
                  true = true,
                  mod.mean = mod.mean,
                  mod.q2.5 = mod.q2.5,
                  mod.q97.5 = mod.q97.5)

# Load library
library(ggplot2)

# Indicate colors for the plot
cols <- c("Truth" = "red", "Estimated" = "black")

# Plot
ggplot(dat, aes(x= names, y=mod.mean))+
  geom_linerange(size = 1, aes(ymin=mod.q2.5, ymax=mod.q97.5)) +
  geom_point(size = 3, aes(x = names, y = mod.mean, col = "Estimated")) +
  geom_point(size = 3, aes(x = names, y = true, col = "Truth")) +
  scale_colour_manual("Values", values=cols)+
  geom_hline(yintercept = 0, lty=2) +
  coord_flip() + ylab('Parameter estimates') +
  xlab("Parameter names") +
  theme_bw()+
  theme(axis.text.x = element_text(size = 17, color = "black"),
        axis.text.y = element_text(size = 17, color = "black"),
        axis.title.y = element_text(size = 17, color = "black"),
        axis.title.x =element_text(size = 17, color = "black"),
        legend.title =element_text(size = 17, color = "black"),
        legend.text =element_text(size = 17, color = "black"),
        panel.grid.major = element_blank(),
        panel.grid.minor = element_blank())
```

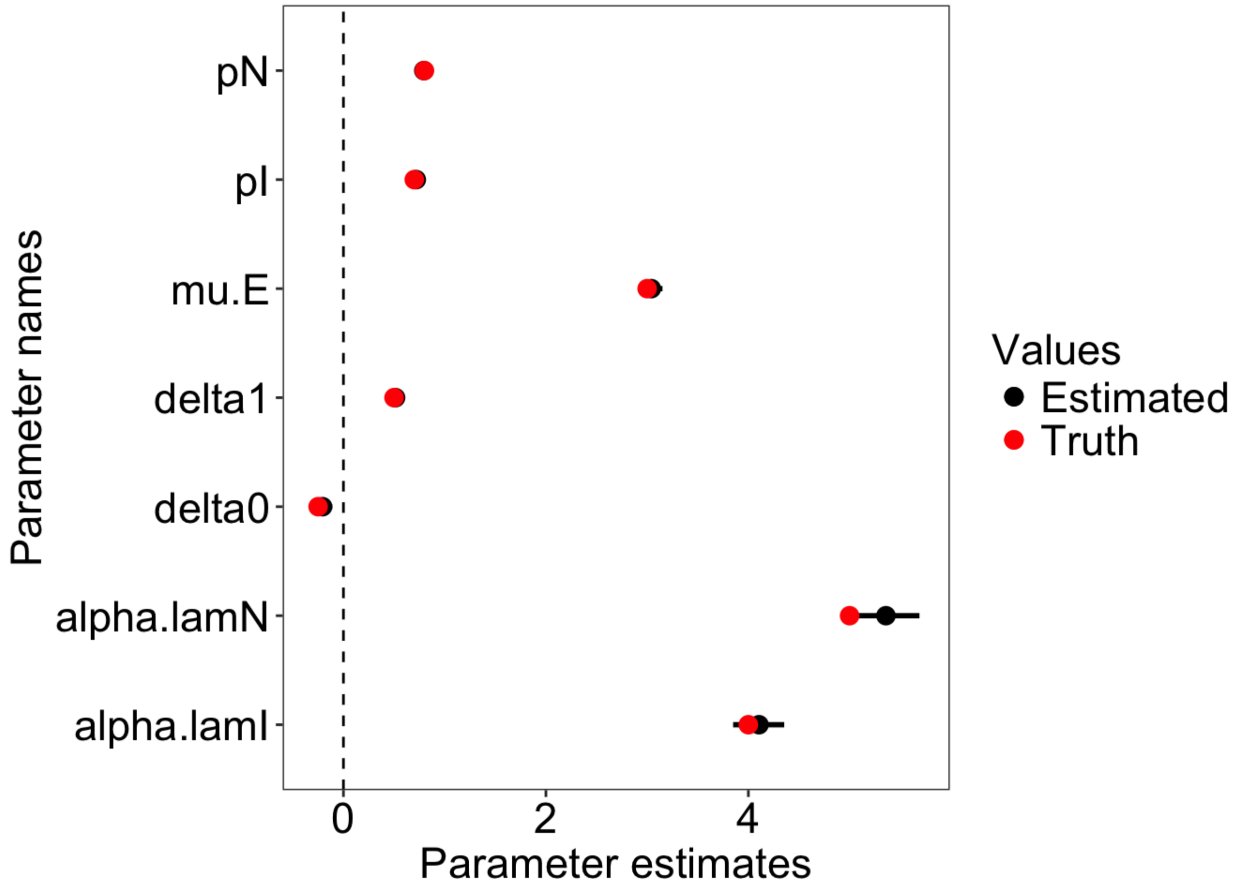

S6: Disease-structured generalized N-mixture model (multi-season)

Abstract- In this section, we aim to demystify the disease-structured generalized N-mixture model by providing a list of state variables, parameters of interest, and code to simulate and analyze data. We highly encourage anyone interested in using this model for their own work or planning a study to use this code as a template for a power analysis. We could use the same formulation as the single season, but we assume that the data were not collected to estimate pathogen detection probability; therefore, we use informative priors.

S6.1: Table

List of state variables, observed data, and parameters of interest along with their definitions.

Quantity	Type	Definition
\(N_{N,i,t}\)	state variable	True total abundance of uninfected hosts at site *i* during season *t*

Quantity	Type	Definition
$N_{I,i,t}$	state variable	True total abundance of infected hosts at site *i* during season *t*
$S_{N,i,t}$	state variable	True abundance of uninfected hosts at site *i* that survived from season *t-1* to *t*
$S_{I,i,t}$	state variable	True abundance of infected hosts at site *i* that survived from season *t-1* to *t*
$G_{N,i,t}$	state variable	True abundance of uninfected hosts at site *i* that were gained from season *t-1* to *t*
$G_{I,i,t}$	state variable	True abundance of infected hosts at site *i* that were gained from season *t-1* to *t*
$T_{N,i,t}$	state variable	True abundance of uninfected hosts at site *i* that transitioned from uninfected to infected from season *t-1* to *t*
$T_{I,i,t}$	state variable	True abundance of infected hosts at site *i* that transitioned from infected to uninfected from season *t-1* to *t*
$Z_{i,j,t}$	state variable	True infection intensity of infected hosts at site *i* during survey *j* and season *t*
$x_{i,j,t}$	Observed data	Observed infection intensity uncorrected for measurement error in sampling or the diagnostic method
$g_{I,i,j,t}$	Observed data	Observed number of infected individuals uncorrected for imperfect pathogen detection
$g_{N,i,j,t}$	Observed data	Observed number of uninfected individuals uncorrected for imperfect pathogen detection
$\alpha.lamI$	parameter	Average abundance of infected hosts at a site the first season
$\alpha.lamN$	parameter	Average abundance of uninfected hosts at a site the first season
p_{I}	parameter	Probability of detecting an infected host
p_{N}	parameter	Probability of detecting an uninfected host
ϕ_{I}	parameter	Probability that an infected individual survives from *t-1* to *t*

Quantity	Type	Definition
ϕ_{N}	parameter	Probability that an uninfected individual survives from *t-1* to *t*
γ_{I}	parameter	Number of infected individual gained at a site from *t-1* to *t*
γ_{N}	parameter	Number of uninfected individual gained at a site from *t-1* to *t*
ψ_{IN}	parameter	Probability of transitioning from infected to uninfected from *t-1* to *t*
ψ_{NI}	parameter	Probability of transitioning from uninfected to infected from *t-1* to *t*
μ	parameter	Average population infection intensity
σ	parameter	Population variation of infection intensity
$\sigma.e$	parameter	Measurement error of the sampling or diagnostic method

S6.2: Simulating data

Write a function to simulate the data.

```
R = 100 # Number of sites
T = 3   # Number of replicate surveys per site
K = 50   # Number of seasons

alpha.lamN = 5   # Average number of hosts per site the first season
alpha.lamI = 3   # Average number of hosts per site the first season
phiN = 0.9
phiI = 0.7  # Apparent host survival probability of uninfected and infect
ed hosts
gammaN = 3 # Average number of individuals arriving at each site for unin
fected hosts
gammaI = 2 # Average number of individuals arriving at each site for infe
cted hosts
pN = 0.8  # Detection probability during each survey
pI = 0.6  # Detection probability during each survey
delta0 = -0.25  # Imperfect pathogen detection intercept (logit scale)
delta1 = 0.5  # Imperfect pathogen detection slope (logit scale)
recovery = 0.1  # Recovery probability
infection = 0.1  # Infection probability
mu = 3
sigma.e = 1.5
sigma = 1.5

# Empty matrices to hold the data
yN <- yI <- array(NA, dim = c(R, T, K))   # Observed abundance data
NI <- NN <- array(NA, dim = c(R, K))      # True abundance data



#-------- First season
  NN[,1] <- rpois(n = R, lambda = alpha.lamN)
  NI[,1] <- rpois(n = R, lambda = alpha.lamI)

#------ Second season
# Empty matrices to hold latent abundance variables
# i.e., number of hosts surviving, arriving, and transitioning

SN <- SI <- GI <- GN <- TrN <- TrI <- array(0, dim = c(R, K-1))

for(k in 2:K){
```

```
  for(i in 1:R){

    if(NN[i,k-1]>0){
      SN[i, k-1] <- rbinom(n=1, size=NN[i,k-1], prob=phiN)
           # Survival of not infecteds
      TrN[i,k-1] <- rbinom(n=1, size=SN[i,k-1], prob=infection)
           # Getting infected
    }
    if(NI[i,k-1]>0){
      SI[i, k-1] <-  rbinom(n=1, size=NI[i,k-1], prob=phiI)
           # Survival of infecteds
      TrI[i, k-1] <- rbinom(n=1, size=SI[i,k-1], prob=recovery)
           # Losing infection
    }
    # Recruitment
    GN[i, k-1] <- rpois(1, lambda = gammaN)
    GI[i, k-1] <- rpois(1, lambda = gammaI)

  }

  # Total
  NI[,k] <-  SI[,k-1] + GI[,k-1] + TrN[,k-1] - TrI[,k-1]
  NN[,k] <-  SN[,k-1] + GN[,k-1] + TrI[,k-1] - TrN[,k-1]

}

# Host obervation process

for(i in 1:R){
  for(j in 1:T){
    for(k in 1:K){
      yN[i, j, k] <- rbinom(n = 1, NN[i,k], pN)
      yI[i, j, k] <- rbinom(n = 1, NI[i,k], pI)
    }
  }
}

# Pathogen observation process
```

```
x <- Z <- m <- theta <- array(NA, dim = c(R, T, K))

for(i in 1:R){
  for(j in 1:T){
      for(k in 1:K){

      # If there were uninfected individuals- assign the average site-lev
el infection intensity
      if(yI[i, j, k] > 0){

        Z[i,j,k] <- rlnorm(1, meanlog = mu, sdlog = sigma)
        x[i,j,k] <- rlnorm(1, meanlog = log(Z[i,j,k]+1), sdlog = sigma.e)

      } else {x[i,j,k] <- NA; Z[i,j,k] <- NA}

      theta[i,j,k] <- plogis(delta0 + delta1 * log(Z[i,j,k]+1))

      m[i,j,k] <- rbinom(1, yI[i,j,k], p = (1-theta[i,j,k]))

    }
  }
}

gN = yN + m
gI = yI - m
```

S6.3: JAGS code

```
{
sink("model.txt")
cat("
model{

# Priors

#------- NOT Infected
alpha.lamN  ~ dnorm(0,0.01) # Average abundance of uninfected hosts the f
irst season
pN     ~ dunif(0,1) # Detection probability of uninfected hosts
gammaN ~ dnorm(0,0.01)  # Gains rate of uninfected hosts
phiN   ~ dunif(0,1) # Survival probability of uninfected hosts
psi_NI ~ dunif(0,1) # Infection probability (transitioning from uninfecte
d to infected)

#------- Infected
alpha.lamI  ~ dnorm(0,0.01)# Average abundance of infecteds the first sea
son
pI     ~ dunif(0,1) # Detection probability of infected
gammaI ~ dnorm(0,0.01)  # Gains rate of infected
phiI   ~ dunif(0,1) # Survival probability of infected
psi_IN ~ dunif(0,1)  # Recovery probability (transitioning from infected
 to uninfected)

#------ Informative priors
delta0 ~ dnorm(-0.25, 0.1)
delta1 ~ dnorm(0.5, 0.1)
tau.e <- 1/(sigma.e * sigma.e)
sigma.e ~ dnorm(1.5, 0.1)
tau <- 1 / (sigma * sigma)
sigma ~ dnorm(1.5, 0.1)
mu ~ dnorm(3, 0.1)

#------------ Ecological model
#---- First season

for(i in 1:R){
  #------ Not infected
```

```
    NN[i, 1] ~ dpois(alpha.lamN)

  #----- Infected
    NI[i, 1] ~ dpois(alpha.lamI)
}

#------ All other seasons
for(k in 2:K){

  for(i in 1:R){

    #------- Nost Infected

    SN[i,k-1] ~ dbin(phiN, NN[i,k-1])  # Total survivors
    TN[i,k-1] ~ dbin(psi_NI, SN[i,k-1])  # Survive, become infected

    GN[i,k-1] ~ dpois(gammaN)   # Recruits

    #------- Infected

    SI[i,k-1] ~ dbin(phiI, NI[i,k-1] ) # Infecteds who survive
    TI[i,k-1] ~ dbin(psi_IN, SI[i,k-1] ) # Get better, transition to unin
fected

    GI[i,k-1] ~ dpois(gammaI)  # Recruits

# Totals

  NN[i, k] <- SN[i,k-1] - TN[i,k-1] + GN[i,k-1] + TI[i,k-1]
  NI[i, k] <- SI[i,k-1] - TI[i,k-1] + GI[i,k-1]  + TN[i,k-1]

  }
}

#------------- Obervation model

for(i in 1:R){
    for(j in 1:T){
      for(k in 1:K){
```

```
#--------- Probability of detecting a host

     yN[i, j, k] ~ dbin(pN, NN[i, k])
     yI[i, j, k] ~ dbin(pI, NI[i, k])

#-------- Probability of detecting the pathogen on a host

    gI[i, j, k] ~ dsum(yI[i, j, k], (-1 * m[i, j, k]))
    gN[i, j, k] ~ dsum(yN[i, j, k], m[i, j, k])

     m[i, j, k] ~ dbin(theta2[i, j, k], yI[i, j, k])
        theta2[i, j, k] <- 1 - theta[i, j, k]
        logit(theta[i, j, k]) <- delta0 + delta1 * log(Z[i, j, k]+1)

    Z[i,j,k] ~ dlnorm(mu, tau)
    x[i,j,k] ~ dlnorm(log(Z[i,j,k]+1), sigma.e)

    }
  }
}

}
", fill = TRUE)
sink()
}
```

S6.4: Analyzing the data

Similar as before, we simulate the data, bundle the data, create initial values, list the parameters to monitor, set the MCMC settings, and run the model.

```
# Bundle data
win.data <- list(gN = gN,
                 gI = gI,
                 x = x,

                 R = dim(yN)[1],
                 T = dim(yN)[2],
                 K = dim(yN)[3])

# Initial values
NIst <- NI
NIst[,-1] <- NA
NNst <- NN
NNst[,-1] <- NA

inits <- function() {list(
  alpha.lamN = alpha.lamN,
  pN = pN,
  phiN = phiN,
  gammaN = gammaN,
  psi_NI = recovery,

  alpha.lamI = alpha.lamI,
  pI = pI,
  phiI = phiI,
  gammaI = gammaI,
  psi_IN = infection,

  delta0 = delta0,
  delta1 = delta1,

  m = m,
  yI = yI,
  yN = yN,

  NN = NNst,
  NI = NIst,

  SN = SN,
```

```
  GN = GN,
  TN = TrN,
  SI = SI,
  GI = GI,
  TI = TrI,

  Z = Z,
  mu = mu,
  sigma = sigma,
  sigma.e = sigma.e

)}

# Monitor Parameters
params <- c("alpha.lamN", "alpha.lamI",
            "pN", "pI",
            "phiN", "phiI",
            "gammaN", "gammaI",
            "psi_NI", "psi_IN"
)
#
### MCMC settings
#ni <- 50000   # Number of iterations
#na <- 10000   # Number of iterations to adapt
#nb <- 20000   # Burn-in to discard
#nt <- 10      # Thinning rate
#nc <- 3       # Number of chains

ni <- 7   # Number of iterations
na <- 1   # Number of iterations to adapt
nb <- 2    # Burn-in to discard
nt <- 1      # Thinning rate
nc <- 3       # Number of chains

library("jagsUI")

out <- jags(win.data, inits, params, "model.txt",
            n.chains = nc, n.thin = nt, n.iter = ni, n.adapt = na,
            n.burnin = nb, parallel = TRUE)
```

```
##
## Processing function input.......
##
## Done.
##
## Beginning parallel processing using 3 cores. Console output will be su
ppressed.
##
## Parallel processing completed.
```

```
## Warning in jags(win.data, inits, params, "model.txt", n.chains = nc,
n.thin
## = nt, : JAGS reports adaptation was incomplete. Consider increasing n.
adapt
```

```
## Calculating statistics.......
##
## Done.
```

Examine the output values and the traceplots to check for covergence and well mixing of chains.

```
print(out)
```

```
## JAGS output for model 'model.txt', generated by jagsUI.
## Estimates based on 3 chains of 7 iterations,
## adaptation = 1 iterations (possibly insufficient),
## burn-in = 2 iterations and thin rate = 1,
## yielding 15 total samples from the joint posterior.
## MCMC ran in parallel for 0.341 minutes at time 2018-11-05 11:19:20.
##
##                    mean      sd      2.5%       50%     97.5% overlap
0 f
## alpha.lamN      4.584   0.188      4.348      4.586      4.893    FALS
E 1
## alpha.lamI      3.111   0.209      2.869      3.064      3.550    FALS
E 1
## pN              0.803   0.001      0.802      0.803      0.805    FALS
E 1
## pI              0.604   0.003      0.599      0.603      0.607    FALS
E 1
## phiN            0.902   0.004      0.896      0.903      0.907    FALS
E 1
## phiI            0.697   0.003      0.693      0.697      0.703    FALS
E 1
## gammaN          3.001   0.044      2.954      2.980      3.070    FALS
E 1
## gammaI          1.961   0.046      1.898      1.960      2.034    FALS
E 1
## psi_NI          0.100   0.001      0.098      0.100      0.102    FALS
E 1
## psi_IN          0.096   0.004      0.090      0.095      0.101    FALS
E 1
## deviance   145818.982 317.661 145335.927 145830.035 146263.109    FALS
E 1
##             Rhat n.eff
## alpha.lamN 0.978    15
## alpha.lamI 1.140    15
## pN         2.763     4
## pI         1.774     5
## phiN       3.583     3
## phiI       1.311     8
## gammaN     6.624     3
```

```
## gammaI     2.199     4
## psi_NI     1.709     5
## psi_IN     2.950     4
## deviance   2.941     4
##
## **WARNING** Rhat values indicate convergence failure.
## Rhat is the potential scale reduction factor (at convergence, Rhat=1).

## For each parameter, n.eff is a crude measure of effective sample size.

##
## overlap0 checks if 0 falls in the parameter's 95% credible interval.
## f is the proportion of the posterior with the same sign as the mean;
## i.e., our confidence that the parameter is positive or negative.
##
## DIC info: (pD = var(deviance)/2)
## pD = 14510.8 and DIC = 160329.8
## DIC is an estimate of expected predictive error (lower is better).
```

```
plot(out)
```

Trace of alpha.lamN

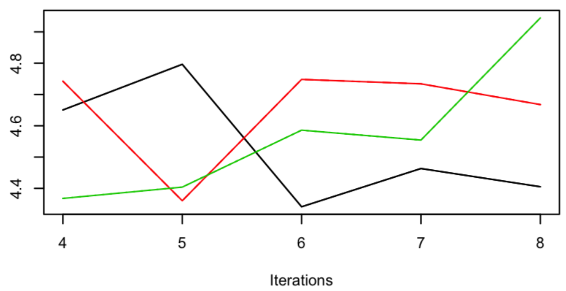

Density of alpha.lamN

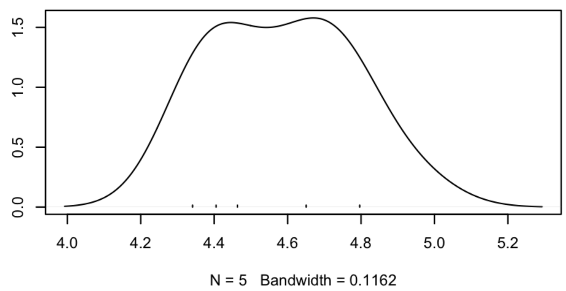

Trace of alpha.lamI

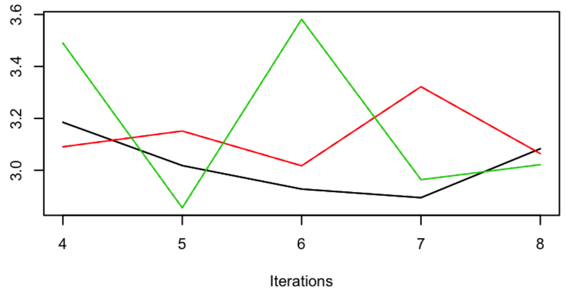

Density of alpha.lamI

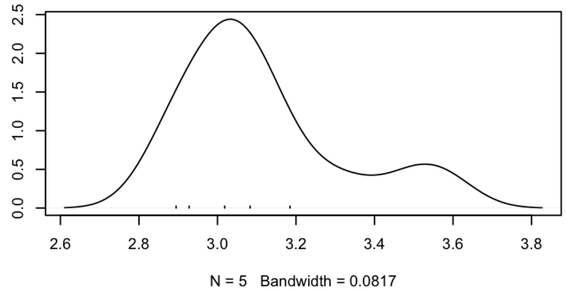

Trace of pN

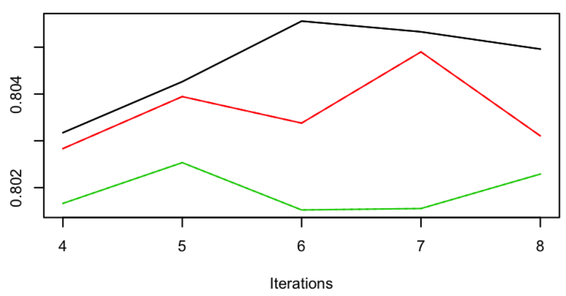

Density of pN

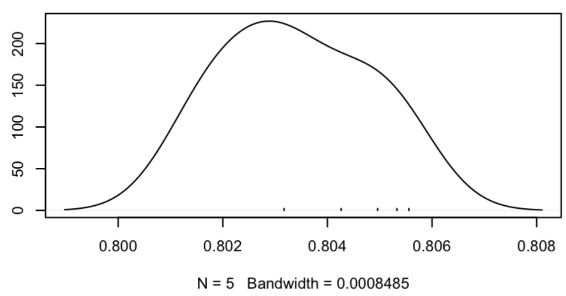

Trace of pI

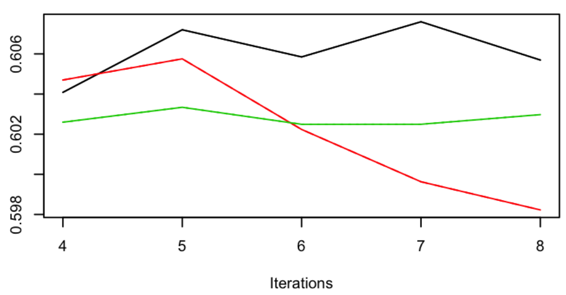

Density of pI

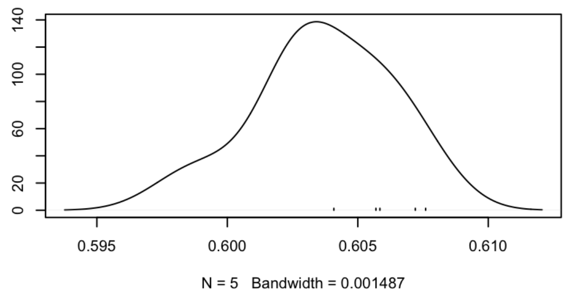

Trace of phiN

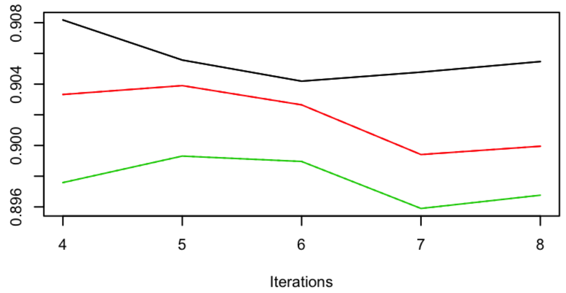

Density of phiN

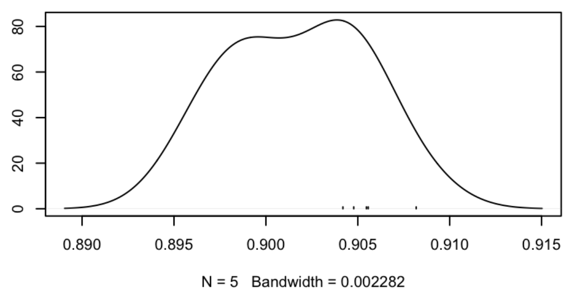

Trace of phiI

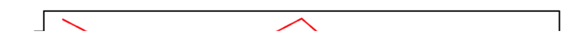

Density of phiI

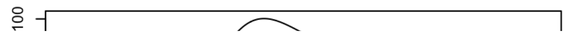

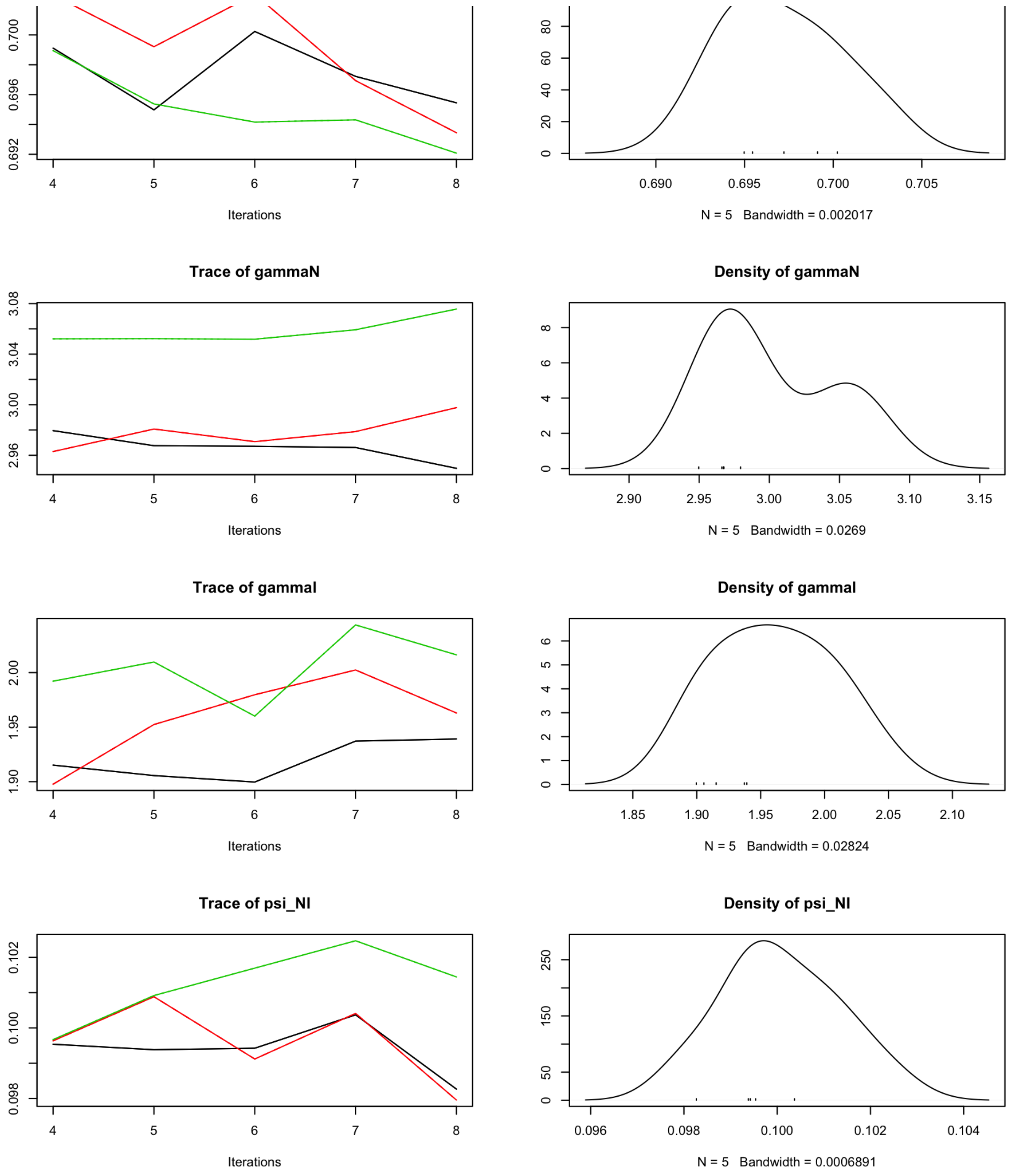

Iterations
N = 5 Bandwidth = 0.002017
Trace of gammaN
Density of gammaN
Iterations
N = 5 Bandwidth = 0.0269
Trace of gammaI
Density of gammaI
Iterations
N = 5 Bandwidth = 0.02824
Trace of psi_NI
Density of psi_NI
Iterations
N = 5 Bandwidth = 0.0006891

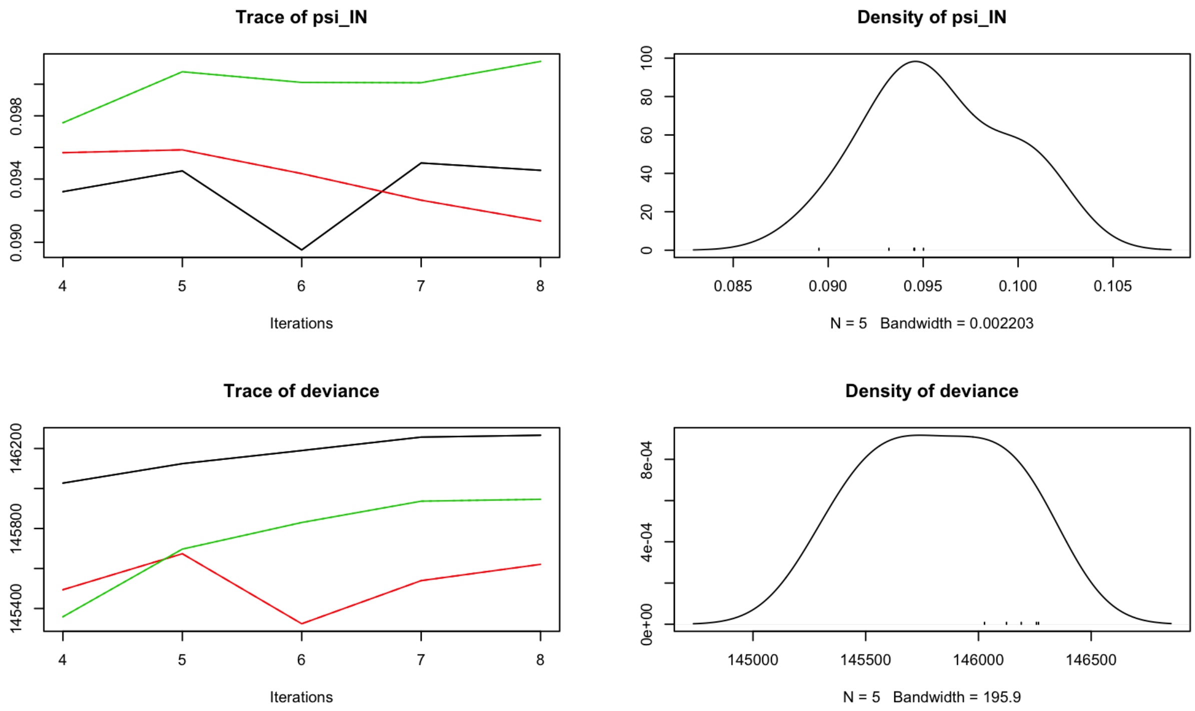

Next, we compare the true parameter estimates to the model estimation.

```
# Store the true paramter values
true <- c((alpha.lamN),
          (alpha.lamI),
          (pN),
          (pI),
          (phiN),
          (phiI),
          (gammaN),
          (gammaI),
          (infection),
          (recovery)
)

# Store the parameter names
names <- c("alpha.lamN", "alpha.lamI",
            "pN", "pI",
            "phiN", "phiI",
            "gammaN", "gammaI",
            "psi_NI", "psi_IN"
)

# Extract the model mean
mod.mean <- c(
  out$mean$alpha.lamN,
  out$mean$alpha.lamI,
  out$mean$pN,
  out$mean$pI,
  out$mean$phiN,
  out$mean$phiI,
  out$mean$gammaN,
  out$mean$gammaI,
  out$mean$psi_NI,
  out$mean$psi_IN)

# Extract the lower credible interval
mod.q2.5 <- c(
  out$q2.5$alpha.lamN,
  out$q2.5$alpha.lamI,
  out$q2.5$pN,
```

```
  out$q2.5$pI,
  out$q2.5$phiN,
  out$q2.5$phiI,
  out$q2.5$gammaN,
  out$q2.5$gammaI,
  out$q2.5$psi_NI,
  out$q2.5$psi_IN)

# Extract the upper credible interval
mod.q97.5 <- c(
  out$q97.5$alpha.lamN,
  out$q97.5$alpha.lamI,
  out$q97.5$pN,
  out$q97.5$pI,
  out$q97.5$phiN,
  out$q97.5$phiI,
  out$q97.5$gammaN,
  out$q97.5$gammaI,
  out$q97.5$psi_NI,
  out$q97.5$psi_IN)

# Combine names, truth, mean, lower CI, and upper CI
dat <- data.frame(names = names,
                  true = true,
                  mod.mean = mod.mean,
                  mod.q2.5 = mod.q2.5,
                  mod.q97.5 = mod.q97.5)

# Load library
library(ggplot2)

# Indicate colors for the plot
cols <- c("Truth" = "red", "Estimated" = "black")

# Plot
ggplot(dat, aes(x= names, y=mod.mean))+
  geom_linerange(size = 1, aes(ymin=mod.q2.5, ymax=mod.q97.5)) +
  geom_point(size = 3, aes(x = names, y = mod.mean, col = "Estimated")) +
  geom_point(size = 3, aes(x = names, y = true, col = "Truth")) +
```

```
scale_colour_manual("Values", values=cols)+
geom_hline(yintercept = 0, lty=2) +
coord_flip() + ylab('Parameter estimates') +
xlab("Parameter names") +
theme_bw()+
theme(axis.text.x = element_text(size = 17, color = "black"),
      axis.text.y = element_text(size = 17, color = "black"),
      axis.title.y = element_text(size = 17, color = "black"),
      axis.title.x =element_text(size = 17, color = "black"),
      legend.title =element_text(size = 17, color = "black"),
      legend.text =element_text(size = 17, color = "black"),
      panel.grid.major = element_blank(),
      panel.grid.minor = element_blank())
```

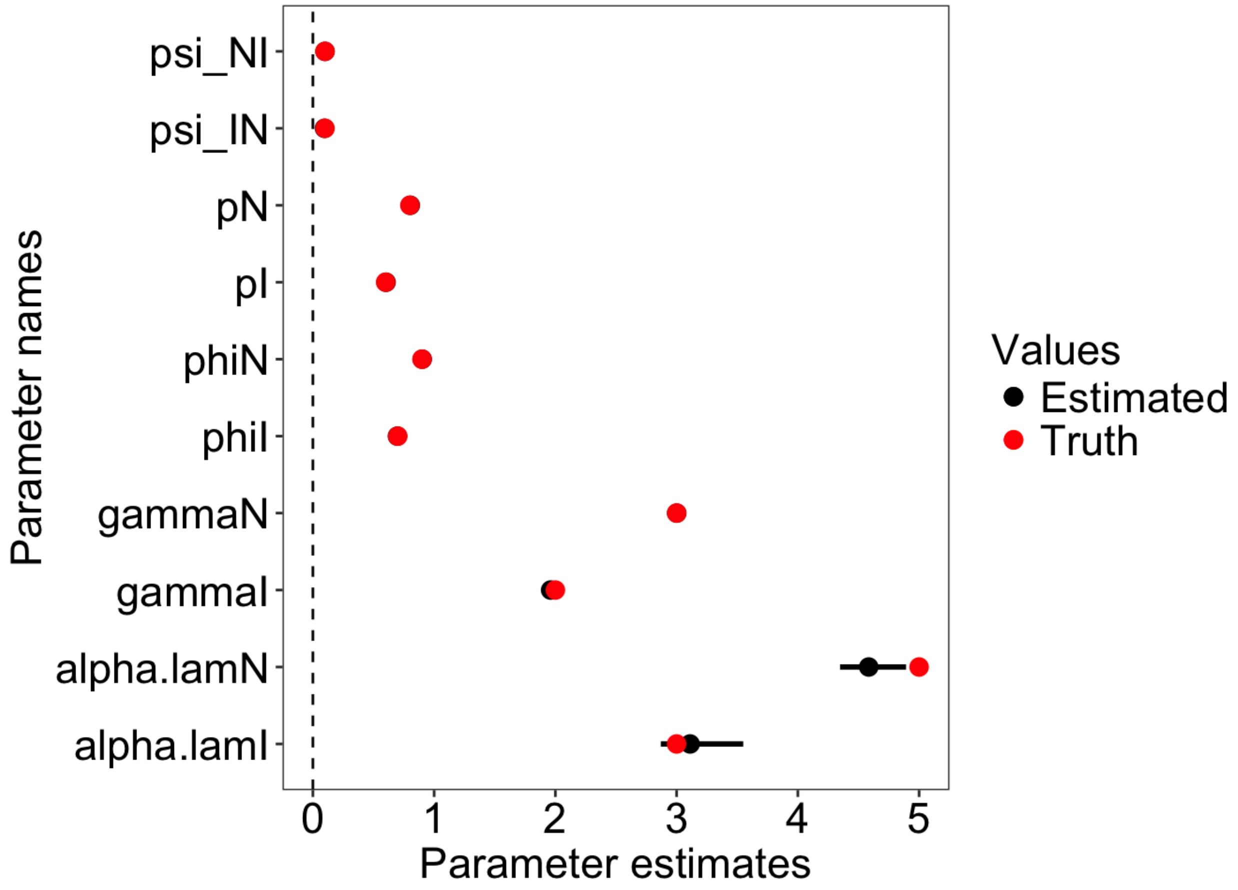

S7: Steps for designing a study and modeling a system

Abstract- This section serves as a practical step-by-step guide for designing a project using unmarked data models outlined in this appendix. We outline the following six steps for any study: (1) Define the study objective, study design, and key model terms, (2) Determine sampling error to consider, (3) Construct a conceptual model addressesing the ecological & sampling processes, (4) Write out the likelihood for the conceptual model, (5) Fit the model to simulated data to isolate mistakes and confirm that model parameters are identifiable, and (6) Collect/compiling the data to analyze and make inference. Following these steps will enable researchers to use unmarked data models for improving inference in the study of disease dynamics. We intend this section to be used simultaneously with the rest of the appendix outlining code, simulating data, and generating models.

Step 1: Define the study objective, study design, and key model terms

Upon defining the study objective(s), define key model terms such as: parameters to estimate (see Table 1 in main text), type of data to collect (i.e., marked or unmarked), sample units (i.e., site definition), the time period over which observations are made and whether populations are assumed closed (i.e., no birth, death, immigration, or emigration) or open (i.e., processes of birth, death, immigration, or emigration), units for replicate surveys (i.e., temporal surveys, spatial surveys, a group of swab samples, multiple qPCR runs, or eDNA samples), and the criterion for ‘detection’ (i.e., evidence of species presence, such as feces or tracks, vs. direct species sighting). Study objectives, logistic or financial constraints, and other study-specific limitations will determine the definition of these design and model parameters. As you work through these definitions and the sampling design, consider the nested structure of your biological system of interest and what role that plays in data collection and analysis (see Fig. 1 in main text). Some of these same principles apply to working with datasets already collected and having a good grasp on study objectives, what the study design was, and the key modeling terms.

For data to be collected, there is a trade-off between the number of sites sampled and the number of replicate surveys per site (MacKenzie & Royle 2005). MacKenzie & Royle (2005) suggest that for rare species, it is more efficient to survey more sampling units less intensively; while for a more common species, fewer sampling units should be surveyed more intensively (if there are no covariates). After completing this step, one should have a solid understanding of what type of model to use and the parameters of interest. Test to ensure that you meet model assumptions in your study design (see Appendix S4). Other model assumptions must be tested after data collection and cannot be controlled in the design phase- although there are variance tradeoffs in accommodating modeling assumption by having a good study design versus during the analysis phase.

Step 2: Determine sampling error to consider

Hosts are regularly missed during survey events because of variations in observer experience, animal behavior, environmental conditions, and random noise (see Fig. 1 in main text; MacKenzie et al. 2006). Just as routinely, pathogens are missed during sampling or diagnostic testing because of imperfect test sensitivity or sampling protocols (Fig. 1 in main text; reviewed in Enoe et al. 2000; Greiner & Gardner 2000; Toft et al. 2005). During a single season, these false negatives result in underestimates of host abundance and pathogen prevalence, and an overestimate of mean infection intensity conditional on infection. Across multiple seasons, imperfect host and pathogen detection can bias host population growth rates, survival and transition probabilities (e.g., Kendall et al. 2004). One way to account for imperfect host and pathogen detection is to repeatedly survey sites over a period of population closure and formulate conditional probability statements in the model. To estimate open population parameters (i.e., birth, death, immigration, emigration), survey the same sites across periods of open dynamics, such as returning to the site the following year/season.

Step 3: Construct a conceptual model addressing the ecological & sampling processes

When constructing a conceptual model of the ecological process for a single season model, consider covariates that influence parameter values, such as site-specific variables that affect host occupancy or abundance. For dynamic or open population models, specify the structure of the host population and the ecological processes, such as birth, death, emigration, immigration, and disease state transitions. By accommodating disease structure in a population, parameters such as recovery and infection probabilities are estimable. These graphical representations (see Fig. 2 in main text) are created using study-system knowledge of the processes affecting host populations and provide the conceptual basis for building mathematical models.

Consider how the observed host states (e.g., seen uninfected, seen infected, not seen) map onto the true host states (e.g., uninfected, infected, dead) when conceptualizing the sampling process. A transition matrix, where rows represent true states and columns refer to observed states, is typically used in multistate mark-recapture models to organize this information. A second question to consider is: “are all states completely observed?” For example, consider the case where an individual is encountered alive, but disease state is not determined (Conn & Cooch 2009; Zipkin et al. 2014*b*). Instead of censoring the data, the observation model can accommodate those data.

Step 4: Use the conceptual model to write the likelihood

Translating the conceptual model to likelihood statements is done for each data type (if multiple are used) or for each process (i.e., ecological vs. observations). The likelihood functions describe the probability of an observed outcome (i.e, the data) conditional on paritcular

parameter values. For example, count data can be analyzed using a linear model, which speficies a gaussian likelihood to estimate abundance and covariate estimates. Typically, the likelihood statements are made using either discrete (e.g., Poisson, negative binomial, etc.) or continuous (e.g., gaussian, gamma, beta, etc.) statistical distributions.

We recommend the following helpful references for learning to translate conceptual models to likelhood statements: MacKenzie et al. 2006, Kéry & Schaub 2012, Kéry & Royle 2016, Royle & Dorazio 2012, Dail & Madsen 2010, Zipkin et al. 2014*a*, *b*, Hobbs & Hooten 2015, Rossman et al. 2016).

Step 5: Fit the model to simulated data to isolate mistakes and confirm parameters are identifiable

After building the likelihood model, we recommend simulating data to confirm that the parameter estimates used to generate the data can be recovered. This ensures that the model was constructed correctly and helps gauge whether the proposed number of sites and replicate surveys are sufficient to adequately estimate parameters of interest as well as providing a power analysis to determine if the strength of covariate signals (e.g., Kéry & Schaub 2012; Kéry & Royle 2016). Adding covariates to the model may increase the amount of data necessary to estimate parameters (Table 1 of main text). Adjust the sampling design as necessary, by either increasing the number of sites sampled or replicate surveys per site. At this phase, it is also important to ensure that the model addresses the study objective and that sufficient data are collected to discriminate among hypotheses.

Step 6: Collect/compiling the data to analyze and make inference

Data collection may be challenging because of uncontrollable circumstances, such as low host recapture rates, preventing the analysis of data as planned. Under this circumstance, it is powerful to have a well-planned study that is amenable to other types of data analyses, such as using unmarked data models.

By using unmarked data models, individual or species detection probabilities have been accomodated, making inference robust and reliable. For example, if the ecological and detection process likelihoods were not teased apart, then covariates that would be significant in the detection process may be significant in the ecological model, leading to erroneous conclusions (Kéry & Schaub 2012).

To quantify parameter effects, we calculate the differences between parameters of interest at each MCMC iteration following Ruiz-Gutiérrez et al. (2010). We compute the proportion of iterations where one parameter is greater than the other. We considered effects with small

posterior medians or with a large degree of parameter overlap to be either unimportant to the process being modeled, or to have been estimated too imprecisely to draw conclusive inference.

S8: Common interfaces and programs to run models

Abstract- It can be difficult to find the balance between model flexiblity, run time, and the learning curve to modeling programs. We provide a list of popular programs used to analyze mark-recapture and unmarked (e.g., detection/non-detection or count) data.

Table

List of common interfaces, names of programs, type of inference, and data needed (i.e., marked vs. unmarked; count vs. detection/non-detection data) to run unmarked data models. This is not an exhaustive list; for example, models can be compiled in C++.

**Inferface/program**	**Type of inference**	**Data input**	**Type of data input**
R; WinBUGS	Bayesian	Marked or unmarked	Count or detection/non-detection
R; OpenBUGS	Bayesian	Marked or unmarked	Count or detection/non-detection
R; JAGS	Bayesian	Marked or unmarked	Count or detection/non-detection
R; NIMBLE	Bayesian/Frequentist	Marked or unmarked	Count or detection/non-detection
R; STAN^{+}	Bayesian	Marked or unmarked	Count or detection/non-detection
R; package *unmarked*	Frequentist	Unmarked data only	Count or detection/non-detection
PRESENCE	Frequentist	Unmarked data only	Detection/non-detection only
MARK	Frequentist/Bayesian	Marked or unmarked	Detection/non-detection only

Inferface/program	Type of inference	Data input	Type of data input
R; package *ednaoccupancy*	Bayesian	Unmarked	Detection/non-detection only

S9: References

Bailey, L. L., MacKenzie, D. I., & Nichols, J. D. (2014). Advances and applications of occupancy models. Methods in Ecology and Evolution, 5, 1269-1279.

Brooks, S.P. & Gelman, A. (1998) General methods for monitoring convergence of iterative simulations. Journal of Computational and Graphical Statistics, 7, 434–455.

Conn, P.B. & Cooch, E.G. (2009). Multi-state capture-recapture analysis under imperfect state observation: an application to disease models. J Appl Ecol, 46, 486-492.

Cooch, E. G., Conn, P. B., Ellner, S. P., Dobson, A. P., & Pollock, K. H. (2012). Disease dynamics in wild populations: modeling and estimation: a review. Journal of Ornithology, 152, S485-S509.

Dail, D., & Madsen, L. (2011). Models for estimating abundance from repeated counts of an open metapopulation. Biometrics, 67, 577-587.

DiRenzo, G. V., Campbell Grant, E. H., Longo, A. V., Che-Castaldo, C., Zamudio, K. R., & Lips, K. R. (2018). Imperfect pathogen detection from non-invasive skin swabs biases disease inference. Methods in Ecology and Evolution, 9, 380-389.

Enoe, C., Georgiadis, M. P., & Johnson, W. O. (2000). Estimation of sensitivity and Sp of diagnostic tests and disease prevalence when the true disease statue is unknown. Preventive Veterinary Medicine, 45, 61-81.

Greiner, M., & Gardner, I. A. (2000). Epidemiologic issues in the validation of veterinary diagnostic tests. Preventive Veterinary Medicine, 45, 3-22.

Hobbs, N. T., & Hooten, M. B. (2015). Bayesian models: a statistical primer for ecologists. Princeton University Press, Princeton, New Jersey, USA.

Kellner, K. (2015). jagsUI: A Wrapper Around ‘rjags’ to Streamline ‘JAGS’ Analyses. R package version 1.3.7. http://CRAN.R-project.org/package=jagsUI (http://CRAN.R-project.org/package=jagsUI)

Kendall, W. L., Langtimm, C. A., Beck, C. A., & Runge, M. C. (2004). Capture-Recapture Analysis for Estimating Manatee Reproductive Rates. Marine Mammal Science, 20(3), 424-437.

Kéry, M., & Royle J. A. (2016) Applied hierarchical modeling in ecology: Analysis of distribution, abundance, and species richness in R and BUGS. Academic Press Inc.

Kéry, M., & M. Schaub. (2012). Bayesian population analysis using WinBUGS. Elsevier, Waltham, Massachusetts, USA.

Lachish, S., Gopalaswamy, A. M., Knowles, S. C. L., & Sheldon, B. C. (2012). Site-occupancy modelling as a novel framework for assessing test sensitivity and estimating wildlife disease prevalence from imperfect diagnostic tests. Methods in Ecology and Evolution, 3, 339-348.

Lunn, D., Jackson, C., Best, N., Thomas, A., & Spiegelhalter, D. (2012) The BUGS book: a practical introduction to Bayesian analysis. CRC Press, Boca Raton, FL.

Kéry, M. (2018). Identifiability in N-mixture models: a large-scale screening test with bird data. Ecology, 99(2), 281–288.

MacKenzie, D. I., Nichols, J. D., Lachman, G., Droege, S., Royle, J. A., & Langtimm, C. 2002. Estimating site occupancy rates when detection probabilities are less than one. Ecology, 83, 2248-2255

MacKenzie, D. I., & Royle, J. A. (2005). Designing occupancy studies: General advice and allocating survey effort. Journal of Applied Ecology, 42, 1105-1114.

MacKenzie, D. I., Nichols, J. D., Royle, J. A., Pollock, K. H., Bailey, L. L., & Hines, J. E. 2006. Occupancy estimation and modeling. Elsevier, Amsterdam.

MacKenzie, D. I., & Royle, J. A. (2005). Designing occupancy studies: general advice and allocating survey effort. Journal of Applied Ecology, 42, 1105-1114.

Miller, D. A. W., Talley, B. L., Lips, K. R., & Campbell Grant, E. H. (2012). Estimating patterns and drivers of infection prevalence and intensity when detection is imperfect and sampling error occurs. Methods in Ecology and Evolution, 3, 850-859.

R Core Team (2018). R: A language and environment for statistical computing. R Foundation for Statistical Computing, Vienna, Austria. URL http://www.R-project.org/ (http://www.R-project.org/).

Royle, J. A. 2004. N-mixture models for estimating population size from spatially replicated counts. Biometrics, 60, 108-115.

Royle, J. A., & Kéry, M. (2007). A Bayesian state-space formulation of dynamic occupancy models. Ecology, 88, 1813-1823.

Toft, N., Jørgensen, E., & Højsgaard, S. (2005). Diagnosing diagnostic tests: Evaluating the assumptions underlying the estimation of sensitivity and specificity in the absence of a gold standard. Preventive Veterinary Medicine, 68, 19-33.

Zipkin, E. F., Thorson, J. T., See, K., Lynch, H. L., Grant, E. H. C., Kanno, Y., et al. (2014*a*). Modeling structured population dynamics using data from unmarked individuals. Ecology, 95, 22-29.

Zipkin, E. F., Sillett, T. S., Grant, E. H. C., Chandler, R. B., & Royle, J. A. (2014*b*). Inferences about population dynamics from count data using multistate models: a comparison to capture-recapture approaches. Ecology and Evolution, 4, 417-426.
